# Ultrasound-triggered therapeutic microbubbles enhance the efficacy of cytotoxic drugs by increasing circulation and tumor drug accumulation and limiting bioavailability and toxicity in normal tissues

**DOI:** 10.7150/thno.49670

**Published:** 2020-09-01

**Authors:** Nicola Ingram, Laura E. McVeigh, Radwa H. Abou-Saleh, Juliana Maynard, Sally A. Peyman, James R. McLaughlan, Michael Fairclough, Gemma Marston, Elizabeth M. A. Valleley, Jorge L. Jimenez-Macias, Antonia Charalambous, William Townley, Malcolm Haddrick, Antonia Wierzbicki, Alexander Wright, Milène Volpato, Peter B. Simpson, Darren E. Treanor, Neil H. Thomson, Paul M. Loadman, Richard J. Bushby, Benjamin R.G. Johnson, Pamela F. Jones, J. Anthony Evans, Steven Freear, Alexander F. Markham, Stephen D. Evans, P. Louise Coletta

**Affiliations:** 1 Leeds Institute of Medical Research, Wellcome Trust Brenner Building, St James's University Hospital, Leeds, LS9 7TF, United Kingdom.; 2 Molecular and Nanoscale Physics Group, School of Physics and Astronomy, University of Leeds, LS2 9JT, United Kingdom.; 3 Department of Physics, Faculty of Science, Mansoura University, Egypt.; 4 Medicines Discovery Catapult, Mereside, Alderley Park, Macclesfield, SK10 4TG, United Kingdom.; 5 Faculty of Electronic and Electrical Engineering, University of Leeds, LS2 9JT, United Kingdom.; 6 Wolfson Molecular Imaging Centre, University of Manchester, Palatine Road, Manchester, M20 3LI, United Kingdom.; 7 Institute of Cancer Therapeutics, University of Bradford, BD7 1DP, United Kingdom.; 8 School of Dentistry, Wellcome Trust Brenner Building, St. James's University Hospital, Leeds, LS9 7TF, United Kingdom.; 9 School of Chemistry, University of Leeds, Leeds, LS2 9JT, United Kingdom.

**Keywords:** Microbubble, ultrasound, VEGFR2, nanoformulation, colorectal cancer

## Abstract

Most cancer patients receive chemotherapy at some stage of their treatment which makes improving the efficacy of cytotoxic drugs an ongoing and important goal. Despite large numbers of potent anti-cancer agents being developed, a major obstacle to clinical translation remains the inability to deliver therapeutic doses to a tumor without causing intolerable side effects. To address this problem, there has been intense interest in nanoformulations and targeted delivery to improve cancer outcomes. The aim of this work was to demonstrate how vascular endothelial growth factor receptor 2 (VEGFR2)-targeted, ultrasound-triggered delivery with therapeutic microbubbles (thMBs) could improve the therapeutic range of cytotoxic drugs.

**Methods:** Using a microfluidic microbubble production platform, we generated thMBs comprising VEGFR2-targeted microbubbles with attached liposomal payloads for localised ultrasound-triggered delivery of irinotecan and SN38 in mouse models of colorectal cancer. Intravenous injection into tumor-bearing mice was used to examine targeting efficiency and tumor pharmacodynamics. High-frequency ultrasound and bioluminescent imaging were used to visualise microbubbles in real-time. Tandem mass spectrometry (LC-MS/MS) was used to quantitate intratumoral drug delivery and tissue biodistribution. Finally, ^89^Zr PET radiotracing was used to compare biodistribution and tumor accumulation of ultrasound-triggered SN38 thMBs with VEGFR2-targeted SN38 liposomes alone.

**Results:** ThMBs specifically bound VEGFR2 *in vitro* and significantly improved tumor responses to low dose irinotecan and SN38 in human colorectal cancer xenografts. An ultrasound trigger was essential to achieve the selective effects of thMBs as without it, thMBs failed to extend intratumoral drug delivery or demonstrate enhanced tumor responses. Sensitive LC-MS/MS quantification of drugs and their metabolites demonstrated that thMBs extended drug exposure in tumors but limited exposure in healthy tissues, not exposed to ultrasound, by persistent encapsulation of drug prior to elimination. ^89^Zr PET radiotracing showed that the percentage injected dose in tumors achieved with thMBs was twice that of VEGFR2-targeted SN38 liposomes alone.

**Conclusions:** thMBs provide a generic platform for the targeted, ultrasound-triggered delivery of cytotoxic drugs by enhancing tumor responses to low dose drug delivery via combined effects on circulation, tumor drug accumulation and exposure and altered metabolism in normal tissues.

## Introduction

A major obstacle to improving cancer outcomes is effective tumor-targeted drug delivery with minimum off-site toxicity. Despite large numbers of anti-cancer drugs being developed, their clinical use is often precluded by poor efficacy, intolerable side-effects and/or drug resistance.

Advanced drug delivery systems developed to overcome these problems, such as liposomes and nanoparticles (NPs), have not yet demonstrated widespread clinical impact. A recent count showed 50 different NPs currently in clinical trials but only 2 were FDA/EMA approved from 2016-2019 1. This is due, in part, to a lack of clear pharmacological evidence of improved efficacy compared with conventional drug delivery and a lack of understanding of the mechanisms by which NP formulations elicit their response. For example, a recent meticulous study showed in multiple tumor types that active transport may be required 2. Using 'Zombie' mice, these researchers showed that active uptake of NPs via transendothelial pathways was predominant, potentially warranting a shift to active tumor targeting and away from the reliance on passive mechanisms involving enhanced permeability and retention (EPR) 2, 3. However, multiple mechanisms including active targeting, EPR and transcytosis 4 could all potentially contribute to effective uptake. Nevertheless, the role of active targeting via antibodies or ligands, is a major area of research in “EPR and beyond” 5 aimed at increasing NP uptake into tumors 6-9. In addition, little understanding of the absorption, distribution, metabolism, excretion and toxicity (ADMET) as well as inadequate tumor accumulation have proved challenging for nanodrug and nanoparticle therapeutics, thus precluding the use of more potent and highly toxic drugs in nanoformulations 10, 11.

To address some of these problems, several actively targeted and triggered systems are being developed where multiplexed particles and external energetic triggers (ultrasound [US], heat, light) or intrinsic tumor triggers (pH, redox) combine for tumor-specific drug delivery 12. This combination approach to anti-cancer drug targeting may provide the required improvement in cancer outcomes with improved quality-of-life using existing or future agents. This is of particular significance when considering cancer incidence and demographics where for example in colorectal cancer (CRC), incidence is strongly related to age and rises steeply from 50-54 years with the highest rates seen in the 85 - 89 age group 13, a group projected to almost double over the next 25 years 14. This population may therefore be more suited to less invasive, low dose or adaptive therapies that control rather than cure cancer.

Microbubbles (MBs) are micron-sized gas-filled phospholipid-shelled spheres, clinically approved for contrast-enhanced US imaging, which also show promise as vehicles for drug delivery 15-17. The first use of MBs for improving tumor drug delivery was via co-injection with free drug with the application of low-intensity US to facilitate drug uptake by “sonoporation”, the formation of pores due to the biomechanical response to oscillating MBs 18-20. Indeed this method has been examined in several clinical trials 21, 22 with some encouraging partial responses in both hepatic metastases and inoperable pancreatic cancer in early Phase I trials. However, more recent pre-clinical studies suggest that the most effective drug delivery by MBs is mediated by liposomes attached to the MB shell 23-25. In this way, US-induced oscillation or collapse of the MB can affect local release and delivery of drug into the target tissue 26. Furthermore, recently described mathematical and single bubble models 27 using fluorescently labelled, targeted MBs bound at a surface, identified physical mechanisms of drug release and transport following US exposure which may involve acoustic streaming, a phenomenon known to be generated by an oscillating bubble close to a border or wall 28.

Due to their micron size, MBs are intra-vascular agents; therefore actively targeting tumor endothelial markers (such as VEGFR2 or α_v_β_3_ integrin) have been used to enhance the imaging of tumor vasculature 29-31 and also to target liposomes 10, 11, 32. VEGFR2 lends itself to this role as it is upregulated in most solid tumors, including CRC 33, 34. Smith *et al.* showed that VEGFR2 (and VEGFR3) was widespread in vessels of malignant tissues of the colon, breast and lung compared with matched normal controls 34. VEGF/VEGFR2 blockade has also proved effective in treating advanced metastatic CRC 35. Molecular imaging with VEGFR2 targeted MBs has been used for monitoring therapy effects in an experimental CRC model 36 and clinically for breast, ovarian, prostate 37, 38 and renal cell cancer imaging 39 demonstrating widespread potential for VEGFR2 in cancer imaging and targeting.

Irinotecan ([1,4'-Bipiperidine]-1'-carboxylic acid, CPT-11) is a chemotherapy drug used in primary and secondary line treatment of CRC (FDA approval given in 1998). Irinotecan is a prodrug, requiring activation by carboxylesterases to form SN38 (7-Ethyl-10-hydroxy-camptothecin), its active metabolite which is up to 1000 times more toxic. SN38 is detoxified by the polymorphic enzyme UGT1A1 to SN38 glucuronide (SN38G) 40. Only a fraction of the administered irinotecan dose is converted to SN38, with the remaining drug being metabolized by CYP3A4 (and possibly CYP3A5) or excreted via hepatic or renal transport 40, 41. SN38 causes cell death by inhibiting topoisomerase I (Topo I), an enzyme vital to DNA replication and transcription 40, 42. This induces irreversible double-strand breaks and subsequently cell death in proliferating cells. Although SN38 shows *in vitro* efficacy as an anti-cancer agent, it is extremely hydrophobic and the fact that it cannot be dissolved in any pharmaceutically acceptable solvent has precluded its direct use.

**Figure 1:**
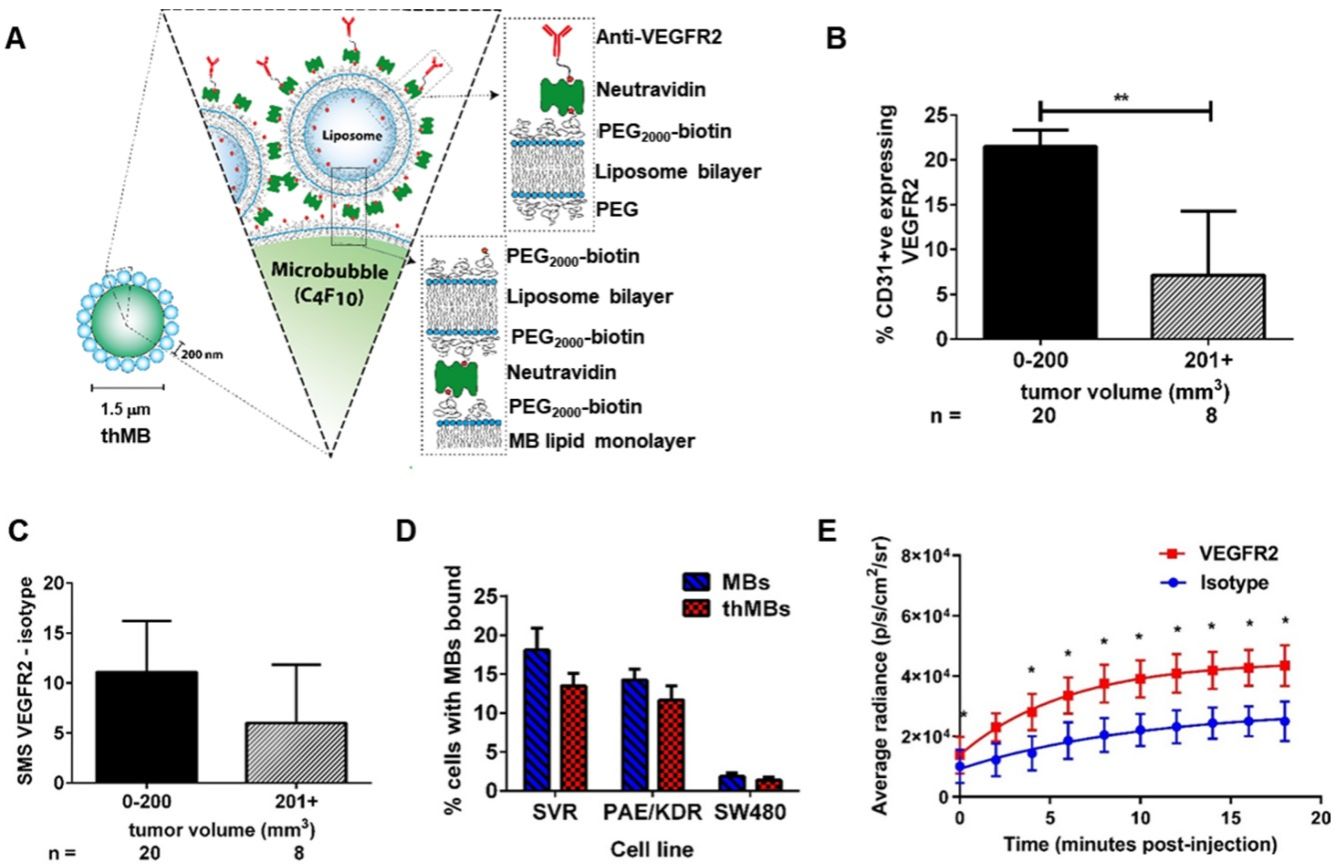
** VEGFR2 targeting of therapeutic microbubbles in human colorectal cancer. (A)** Schematic depiction of a thMB **(B)** HCT116 CRC xenografts immunostained for CD31 and VEGFR2 with percentage of VEGFR2/CD31 double positive blood vessels related to tumor size presented (median with 95% confidence levels are denoted, **p = 0.004, Mann-Whitney U Test, two tailed). **(C)** The SMS (HF-US signal from VEGFR2-bound MBs minus the HF-US signal from isotype-control MBs) binned against tumor volume shows the specific binding of VEGFR2-targeted MBs *in vivo* (median with 95% confidence levels are denoted, not statistically significantly different). **(D)** Targeted microbubbles (MBs) or thMBs both with VEGFR2 targeting were flowed over SVR or PAE/KDR endothelial cells (both VEGFR2-expressing) and SW480 CRC cells (non-VEGFR2 expressing). The percentage of cells with MBs bound was calculated (see 'Quantification of VEGFR2-targeted MB binding' in the Methods section). Mean and SEM are plotted. Two-way repeated measures ANOVA comparing between the MB type within the same cell type shows no significant differences in binding. **(E)** Bioluminescent imaging of mice injected with either isotype-targeted luciferin MBs or VEGFR2-targeted luciferin MBs. The average radiance measured from the pairs of mice are shown (*n* = 5 pairs). Fitted with a one-phase association curve, paired t-tests, one tailed at each time point *p* < 0.05 for all. The curve-fitted mean and SEM are shown (R^2^ isotype = 0.981, R^2^ VEGFR2 = 0.998).

We have previously developed a flexible microfluidic MB production platform 43 that allows on-chip assembly of therapeutic MBs (thMBs) (see **Figure 1A** for schematic) of defined size and lifetime 44 and showed US-triggered model drug release from liposomally-loaded MBs *in vitro* 45, 46. In this study we have engineered thMBs with either the prodrug irinotecan or its highly toxic metabolite SN38 and investigated their biodistribution, efficacy and mechanisms of action *in vivo*. Using this approach, we have shown that thMBs can deliver effective doses of highly toxic drugs to tumors resulting in tumor growth inhibition without toxic side effects and may therefore provide the means to formulate existing potent but toxic drugs, novel drug combinations or previously “hard to deliver” drugs for cancer therapy.

## Methods

### Reagents and materials

Lipids used for MB and liposome production were from Avanti Polar Lipids (Alabaster, AL, USA). Texas Red® 1,2-dihexadecanoyl-*sn*-glycero-3-phosphoethanolamine, triethylammonium salt (Texas Red® DHPE), tissue culture reagents and neutravidin were all from Invitrogen, Life Technologies (UK). Cardiolipin, sucrose, manganese sulfate, 4-(2-hydroxyethyl)-1-piperazine ethanesulfonic acid (HEPES), ethylenediamine tetraacetic acid (EDTA), dimethyl sulfoxide (DMSO), irinotecan hydrochloride, ethyl-10-hydroxycamptothecin (SN38), tolbutamide and calcium ionophore A23187 were all from Sigma-Aldrich (St Louis, MO, USA). 7-ethyl-10-(4-amino-1-piperidino) carbonyloxycamptothecin (SN38G) and irinotecan-d10 hydrochloride were from Santa Cruz Biotechnology Inc. (Dallas, Texas, USA). Luciferin was purchased from Promega (Madison, WI, USA). 2,5-dioxopyrrolidin-1-yl3-oxo-1-(phenyldisulfanyl)-7,10,13,16,19,22,25,28,31,34,37,40,43,46,49,52-hexadecaoxa-4-azapentapentacontan-55-oate (SPDP-dPEG1k-NHS ester) was purchased from Quanta Biodesign Ltd (US) and *N*-(3-Maleimido-1-oxopropyl)-L-α-phosphatidylethanolamine, Distearoyl (DSPE-Maleimide) was purchased from NOF Europe GmbH (Germany). ^89^Zr-oxalate was purchased from Perkin Elmer (US)/BV cyclotron (Netherlands). All other chemicals were purchased from Sigma Aldrich (UK) and were used without any further purification.

Jupiter® 10 µm Proteo(C12) 90 Å, LC Column 250 × 10 mm and Jupiter® 10 µm C4 300 Å, LC Column 250 × 10 mm HPLC columns were purchased from Phenomenex LTD (UK) while a Superdex-200 10/300 HPLC column was purchased from Sigma Aldrich (UK). PD-10 size exclusion desalting columns were purchase from GE Healthcare (UK) and Amicon ultra-centrifugal filters were purchased from Sigma Aldrich (UK).

HPLC analysis was performed on a Shimadzu Prominence LC system running Laura 3 software from LabLogic and an ALC PK121R centrifuge was used as part of the ^89^Zr-liposome purification/filtration process.

### Cell lines

SW480, SW620 and HCT116 human CRC cell lines were obtained from ECACC (ecacc.org.uk). MC38 luc11A mouse syngeneic CRC cells, expressing luciferase were a kind gift from Professor R. D. Beauchamp, University of Vanderbilt, USA under a Materiasl Transfer Agreement. SVR murine endothelial cells were purchased from ATCC. Porcine aortic endothelial cells transfected with the human VEGFR2 gene (PAE/KDR) were a kind gift from Professor L Claesson-Welsh, University of Uppsala, Sweden. Cells were maintained in RPMI with 10% (v/v) fetal calf serum (FCS, Sigma-Aldrich, St Louis, MO, USA), apart from the PAE/KDR cells which were cultured in Ham's F12 medium supplemented with 10% FCS. The SVR cells were cultured in high-glucose DMEM with 5% FCS. All cells were maintained at 37°C in 5% CO_2_. All human cell lines were authenticated in-house by tandem repeat (STR) profiling and screened negative for mycoplasma.

### Mouse models

All experiments were performed following local ethical approval and in accordance with the UK Animals (Scientific Procedures) Act 1986. CD-1® nude mice were bred in-house under license from Charles River Laboratories (Wilmington, MA, USA) and maintained in specific-pathogen free conditions in individually ventilated cages (IVCs) with free access to water and food.

Human CRC cells were used to establish xenografts on the flank of 5-7 week old female CD1 nude mice, as described previously 47. The numbers of mice (*n*) per group are detailed in each figure.

### High-frequency ultrasound imaging (HF-US)

Tumors were imaged using a Vevo 770 high-frequency ultrasound system (Fujifilm VisualSonics Inc., Ontario, Canada) with 40 MHz (RMV-704) and 25 MHz (RMV-710B) transducers, as previously described 48.

Contrast enhanced US and quantitative molecular imaging with US destruction-replenishment and Target-Ready Micromarker (Fujifilm VisualSonics Inc., Ontario, Canada) were performed as previously described 31 using 27g tail vein catheters (SAI Infusion Technologies, IL, USA) and an infusion syringe pump (Aladdin, World Precision Instruments, UK).

### Bioluminescence imaging (BLI)

Light microscopy was used to measure the concentration and mean diameter of MBs and DLS was used to measure the mean diameter of liposomes. Using these parameters, we calculated that 100 µL liposome-loaded MBs (Target-Ready MicroMarker), corresponding to 1 × 10^8^ microbubbles carried approximately 340 liposomes per MB. This was injected via the tail vein into MC38 luc11A tumor bearing mice. MBs were targeted using biotinylated anti-VEGFR2 antibody or isotype control antibody as described 31. Pairs of mice with similar-sized tumors were injected via tail vein catheters to allow simultaneous imaging of targeted and untargeted liposomes. Auto exposure settings on an IVIS Spectrum (Perkin Elmer, Walton, MA, USA) were used for *in vivo* imaging (maximum one min exposure time) and BLI was performed every two min. Living image software version 4.4 was used to analyze BLI signals. Auto ROIs with a threshold of 14% were used to calculate average radiance (photons/second/cm^2^/steradian).

### Quantification of VEGFR2-targeted MB binding

*In vitro* binding efficiency of microfluidically-generated VEGFR2-targeted MBs and thMBs (see On-chip generation of thMBs for details) was assessed as previously described 43, using flow rates of 0.3 - 0.6 mL/min. The percentage of cells with MBs bound was calculated using the following equation:


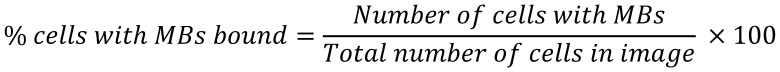


### Generation and characterization of luciferin liposomes

Luciferin encapsulation into liposomes was performed by active acetate gradient loading through the liposome membrane 49. The shell was composed of 56:4:40:0.1 mol% of DSPC:DSPE-Biotin-PEG_2000_:Cholesterol:Texas Red® DHPE.

Concentrations and size distributions of liposome preparations were determined using a Q-nano instrument (Izon Science Ltd, Oxford, UK). The encapsulated concentration of luciferin was determined using an LS55 Fluorescence Spectrometer (Perkin Elmer Inc., UK) against a calibration curve of free luciferin.

### Generation and characterization of irinotecan liposomes

The lipid shell was composed of 63:32:5:0.1 mol% DSPC:Cholesterol:DSPE-Biotin-PEG_2000_:Texas Red® DHPE. Irinotecan encapsulation was performed by the manganese sulfate pH gradient method, as previously described 50. Liposomes were sized and their concentration determined by Q-nano (Izon Science, Oxford, UK). The stability of liposomes was examined by measuring the irinotecan concentration, size distribution and liposome concentration for a sample that was stored at 4 °C over a period of 8 weeks. Encapsulated irinotecan was quantified by HPLC (see below for HPLC methods).

### Generation and characterization of SN38 liposomes

The lipid shell of SN38 liposomes was comprised of 57:39:4:0.1 mol% of DSPC:Cholesterol:DSPE-Biotin-PEG_2000_:Texas Red® DHPE. 11% by weight of cardiolipin was also added to the shell. SN38 was dissolved in 0.1 M ammonium hydroxide to 2 mg/mL and the encapsulation procedure was carried out as previously described 51. Liposomes were frozen in aliquots in liquid nitrogen, dehydrated by freeze drying then stored at 4 °C until required. Samples were rehydrated prior to use with 10 mM acetate buffer, pH 2 and filtered through a 200 nm filter. The encapsulated concentration of SN38 was measured by tandem mass spectrometry (see below for details). Liposomes were sized and their concentration was measured by DLS (Malvern) and NanoSight (Malvern). A Zetasizer (Malvern Instruments Ltd, UK) was used to measure the zeta potential, by passing a voltage through the sample and mixed measurement model phase analysis (M3-PALS) technology determined the mean reading in mV. The zeta potential was determined as -50.2 ± 7.7 mV, suggesting these liposomes would have long term stability with minimal aggregation.

### Synthesis of Df-PEG1k-SPDP

The synthesis of Df-PEG1k-SPDP was carried out according to the method described 52. First deferoxamine mesylate (39 mg, 60 µmol) was dissolved in DMSO (1 mL) and SPDP-PEG1k-NHS (65 mg, 60 µmol, in DMSO (1 mL)) and diisopropylethylamine (7.8 mg, 60 µmol, 10.5 µL) were added. The reaction solution was stirred at room temperature for 4 h and monitored by HPLC (Jupiter® 10 µm Proteo (C12) 90 Å, Column, 250 x 10 mm). Upon completion the reaction mixture was poured into deionized water (0.5 mL) and the product was isolated by HPLC with an eluent system of solvent A = water (0.05 % TFA) and solvent B = acetonitrile (0.05 % TFA) running at a gradient of 10 to 60 % solvent B over 30 min at a flow rate of 3 mL/min with a UV detector wavelength of 220 and 280 nm.

### Synthesis of Df-PEG1k-DSPE

The synthesis of Df-PEG1k-DSPE was carried out according to the method described 52. DSPE-maleimide (15 mg, 16 µmol) was suspended in distilled water (0.5 mL) and sonicated at 50 ºC until the solution became clear before being cooled to room temperature. Df-PEG1k-SPDP (46 mg, 30 µmol) from the previous step was then dissolved in distilled water (1 mL) and the pH was adjusted to 7.3 with NaOH (1 M). TCEP (0.1 M, pH 7, 1 mL) was added to the Df-PEG1k-SPDP solution and the mixture was incubated at room temperature for 10 min. Both solutions, Df-PEG1k-SPDP and DSPE-maleimide were combined and the pH was re-adjusted to 7.0 -7.3 before the reaction mixture was incubated at room temperature for 4 h. After incubation the pH of the reaction mixture was adjusted to pH 2 - 3 and Df-PEG1k-DSPE was isolated by HPLC (Jupiter® 10 µm C4 300 Å, column 250 × 10 mm) with an eluent system of solvent A = water (0.05% TFA) and solvent B = acetonitrile (0.05% TFA) running at a gradient of 50 to 90 % solvent B over 40 min at a flow rate of 3 mL/min with a UV wavelength of 220 and 280 nm.

### Incorporation of Df-PEG1k-DSPE onto liposomes

The lipid shell of SN38 liposomes was comprised of 57:39:4 mol% of DSPC:Cholesterol:DSPE-Biotin-PEG_2000_. 11% by weight of cardiolipin was also added to the shell along with 1 mol% of Df-PEG1k-DSPE.

### Zirconium-89 radiolabeling of Df modified liposomes

First, freeze dried Df-modified liposomes were reconstituted in 10 mM sodium acetate buffer, pH 2 (250 µL) before being passed through a 200 nm syringe filter. Next ^89^Zr-oxalate (230 µL, approx. 220 MBq) was neutralized by the addition of Na_2_CO_3_ (2 M, 105 µL) and the mixture was incubated at room temperature for 5 min. Following the incubation HEPES buffer (0.5 M, pH 7, 1 mL) and the filtered Df-modified liposomes (200 µL) were added. The radiolabelling solution was incubated at room temperature for 60 min followed by the addition of EDTA (0.1 M, 100 µL). Next the reaction mixture was applied to a PD-10 size exclusion purification column and ^89^Zr-liposomes were eluted in phosphate buffered saline (PBS). Finally the ^89^Zr-liposome mixture was filtered on a 100 kDa MWCO centrifuge filter at 12000 rpm for 20 min to give ^89^Zr-liposomes in approximately 0.4 mL of PBS. For quality control analysis 50 µL of the purified ^89^Zr-liposomes were injected onto a Superdex 200 size exclusion column eluted with PBS at 0.8 mL/min. Df modified liposomes were successfully radiolabelled with ^89^Zr to give 41.2 ± 10.8 MBq (*n* = 3) of ^89^Zr-liposomes in a non-decay corrected radiochemical yield of 18.7% ± 4.9 (*n* = 3). The radiochemical purity of ^89^Zr-liposomes was 87.1% ± 0.8 (*n* = 3) as determined by size exclusion HPLC.

### On-chip generation of therapeutic MBs

The generation of thMBs was carried out in a microfluidic device as previously described 43, 44. Briefly, 8 μL 2.5 mg/mL neutravidin was added per 100 μL of liposomes and incubated for 20 min. This was then incubated with 2 mg/mL DPPC, DSPE-biotin PEG200 (95:5 mol%) lipid and 10 µL/mL C_6_F_14_ liquid was added (Sigma-Aldrich, St Louis, MO, USA) to improve MB stability 44 before generating liposome-loaded microbubbles using a patented microfluidic chip-based system. The microbubbles were counted and sized by taking light microscopy images of the resulting microbubbles and 0.1 μg of biotinylated anti-mouse VEGFR2 antibody was added per 10^7^ microbubbles and incubated for 20 min before injection.

### *In vivo* delivery and ultrasound triggering

CD1 nude female mice aged 5-7 weeks were inoculated with 10^7^ SW480 CRC cells on the right hind flank. After 7 days, mice were randomly assigned to a treatment group. ThMBs or free drug were administered via tail vein injection. Animals in the thMBs + trigger (T) group received an US pulse at the tumor site 4 min post MB injection using a custom-built single element ultrasound system (UARP) 45. A 2.2 MHz, 10 µs 'tone burst' US pulse was generated by an unfocused transducer (V323, Olympus NDT, UK), with a peak negative pressure of 260 kPa that had a 1 kHz pulse repetition frequency (PRF). The total sonication time was 5 s. The US-trigger had an MI of 0.21 by Church approximation 53 and a thermal index in soft tissue of 0.09, both of which are considered safe for all diagnostic US applications.

### High-performance liquid chromatography (HPLC)

For quantification of irinotecan loading into liposomes and final MB preparations, HPLC was used. Irinotecan was dissolved in DMSO to 2 mg/mL and a 6-point standard curve diluted 1:1 from 1 mg/mL was prepared in methanol. thMB samples were diluted 1:1 in methanol, vortex mixed and centrifuged at 10,000 g for 4 min at 4°C to remove lipids. Irinotecan liposomes alone were diluted 1:10 in methanol then treated as the thMB sample. Samples were analyzed using a Waters Alliance 2695 HPLC (Waters Corporation, Milford, MA, USA) linked to a Waters 2996 Photodiode Array Detector detector. 10 µL of sample was eluted using a stepwise gradient at a flow rate of 1 mL/min on a Hichrom RPB column (3.5 µm, 25 cm × 2.1 mm) (Hichrom Limited, UK). Mobile phase A consisted of 90% (v/v) dH_2_0, 10% (v/v) methanol and 0.1% (v/v) formic acid, and mobile phase B consisted of 90% (v/v) methanol, 10% (v/v) dH_2_0 and 0.1% (v/v) formic acid. The initial gradient of 90% A: 10% B was gradually increased to 90% B over 7 min, held for 2 min and returned to the initial gradient over 1 minute and held at this to give a total run time of 15 min. The area under the curve at 373 nm was calculated using Empower Pro software (Waters) and comparison to the standard curve was used to determine concentration of irinotecan in liposomes and in each MB preparation. These mobile phases allowed direct conversion to mass spectrometry.

### Tandem mass spectrometry (LC-MS/MS)

Irinotecan, SN38 and SN38G were dissolved in DMSO as fresh 1 mg/mL stock solutions and a 6-point standard curve diluted 1:1 from 1 µg/mL was prepared in methanol. Mouse tumors and tissues were weighed and homogenized in methanol on ice using an Ultra Turrex^®^ blender (Janke and Kunkel, IKA). A clear supernatant was obtained by centrifugation at 10,000 g for 4 min at 4 °C and analyzed by LC-MS/MS. An internal standard (irinotecan-d10 hydrochloride) was also prepared in methanol and spiked into each sample at a concentration of 1 µg/mL. Multiple reaction monitoring (MRM) mass spectrometry was performed on a Waters Quattro Ultima triple quadruple mass spectrometer with an electrospray ionization source operating in positive ionization mode (LC-MS/MS). Compounds were eluted using a stepwise gradient at a flow rate of 0.3 mL/min where mobile phase A and B were as described for HPLC above, on an Acuity UPLC BEH C18 column (1.7 µm, 2.1 × 100 mm) (Waters Corporation, Milford, MA, USA). The column was heated to 40 °C. The initial gradient of 80% A: 20% B was gradually increased over 15 min to 80% B, then increased over 1 min to 100% B and held for 4 min then returned to the initial gradient over 1 min and held for 14 min, with a total run time of 35 min. Instrument settings: Capillary voltage, 3.50 kV; Cone energy, 12 V; Source temperature, 120 °C; Desolvation temperature, 250 °C; Gas flow desolvation, 650 L/h; Cone, 60 L/h. MRM settings were optimized using pure irinotecan and SN38 as described previously 54, The peak area calculated using Masslynx software (Waters Corporation) and comparison to the standard curve was used to determine drug concentrations.

For quantification of SN38 loading into liposomes and final MB preparations, LC-MS/MS was used. Samples were prepared as previously stated for irinotecan liposomes and MBs and analyzed using the LC-MS/MS conditions stated below for SN38 tissue samples.

Tissues were weighed and homogenized in methanol spiked with 10 ng/mL internal standard tolbutamide (Sigma-Aldrich) using a Bead Ruptor 24 Bead Mill Homogenizer (OMNI International Inc.) with 2.8 mm zirconium ceramic oxide beads. A clear supernatant was obtained by centrifugation at 10,000 g for 4 min at 4 °C. Standard curves for SN38 and SN38G were prepared as described previously with tolbutamide spiked methanol, and analyzed by LC-MS/MS using mobile phases and column as described above. The initial gradient of 80% A: 20% B was gradually increased over 16 min to 100% B then returned to the initial gradient over the following 4 min and held, for a total run time of 25 min. Instrument and MRM settings were as described above.

### Positron Emission Tomographic Imaging of radiolabelled liposomes and therapeutic MBs

SW480 tumor bearing CD1 nude female mice were injected intravenously with 100 μL of either ^89^Zr-labelled SN38 liposomes (*n* = 15 mice) or thMBs carrying the ^89^Zr- labelled SN38 liposomes (*n* = 15 mice). Both were conjugated to anti-VEGFR2 antibodies. The injections contained the same number of radiolabeled liposomes per MB (~340) and at 2 MBq per injection. The therapeutic MB group was also subject to an US-trigger as described in '*In vivo* delivery and ultrasound triggering'. Imaging was performed using the Inveon Multimodality TM PET scanner (Siemens Medical Solutions) for 20 min in a supine position after 1, 24 and 72 h post-injection. Five mice per time point were imaged and had their tissue collected after imaging. Data were acquired using Inveon Acquisition Workplace (IAW) software (Siemens) version 2.1 and analyzed using Inveon Reconstruction Workplace (IRW) software (Siemens) version 2.2.0. Images were reconstructed using the order subset expectation maximization (OSEM)/maximum a posteriori (MAP) algorithm.

Tissues were weighed and were counted in a gamma counter (Perkin Elmer, 1480, Wizard 3) for 20 s per sample. The gamma counter provides a “counts per minute” parameter. These data were imported into an Excel spreadsheet where the counts per minute were converted into activity by conversion into disintegrations per minute; by multiplying the efficiency of the gamma counter for ^89^Zr. Activity was decay corrected to the time of injection and converted into a concentration using the *ex vivo* tissue weight (in kilobecquerel per gram). All mice in which ^89^Zr radioactivity in the tail exceeded 10% of the injected dose were excluded from analysis.

### Tissue processing, immunohistochemistry and pharmacodynamic analysis

Paraformaldehyde-fixed tumors and tissues were processed using a tissue processor and embedded in paraffin. 4 µm central sections were cut and one section from each sample was stained with hematoxylin and eosin (H and E). Slides were scanned using an Aperio digital slide scanner (AT2, Leica Biosystems) at ×20 magnification with ImageScope software (Leica Biosystems,) at 0.5 µm/pixel. JPEG compression quality 70 was used to quantify digital slide images for mitotic bodies, which were counted by a reviewer blinded to the experimental groups. ImageScope was then used to manually annotate the boundary of the tumor area on the digital slide, in order to calculate the number of mitoses/mm^2^.

IHC detection of CD31-positive blood vessels was performed as previously described 55 except heat-mediated antigen retrieval in citrate buffer, pH 6.0 was used. Slides were digitally scanned at ×20 magnification to facilitate image analysis and scoring. For quantification of CD31 positive blood vessels, ten 0.25 mm^2^ boxes were placed randomly across the tumor section using RandomSpot software version 6.02 56 and the number of CD31-positive vessels counted as described 57.

VEGFR2-positive blood vessels were identified and quantified in tissues using immunostaining with an anti-mouse VEGFR2 antibody (55B11, Cell Signaling Technologies). Sections were de-paraffinised before heat-mediated antigen retrieval in Tris/EDTA/Tween-20 (10 mM/1 mM/0.05%) buffer pH 9.0. Endogenous peroxidases and casein were also blocked, and then sections were incubated with rabbit anti-mouse antibody at 1:100 dilution for one hour at room temperature. After washing, a rabbit Envision-HRP polymer and DAB were used for visualization (Dako, United Kingdom). Slides were digitally scanned at ×20 magnification. VEGFR2 positive vessels in each tissue section were counted by a blinded observer.

Cleaved Caspase 3 staining of the tumors as a biomarker of apoptosis 47 and phosphorylated histone H2AX (pH2AX) staining were carried out manually as described, except the latter antibody was applied at a 1:400 dilution 58. pH2AX was used as a surrogate marker for irinotecan efficacy, as topoisomerase 1 inhibition causes single-strand DNA breaks, which are then converted to double-strand breaks during S phase. The entire tumor area was digitally scanned at ×20 magnification. Annotated tumor boundaries were used as regions of interest for automated analysis. An in-house developed algorithm was used to extract each region and block process the tissue at full resolution, using color deconvolution to separate staining channels into hematoxylin and DAB images, for analysis independently 59. The intensity of DAB staining was co-localized with a basic binary mask of foreground tissue (generated by thresholding of hue, saturation and intensity channels), in order to obtain the total percentage of positive pixels within the tumor area. The resulting percentage positive pixels were divided by the tumor area, as described previously.

### Transmission electron microscopy (TEM)

Liposomes and thMB morphology was evaluated using negative staining technique with transmission electron microscope (TEM) JEM1400, 120KV instrument (JEOL, USA). For sample preparation, 8 µL of diluted liposome sample (10^11^/mL) or thMB sample (10^9^/mL) was placed on a coated carbon grid. The solution was left on the grid for 30 s, and the excess liquid dried out with filter paper. Another 8 µL of 1% uranyl acetate was subsequently added to stain the sample, incubated for 15 s, then the excess liquid was removed with filter paper, and was finally dried at room temperature.

### Quantitating perivascular inflammation

For perivascular inflammation scoring in the livers of treated animals, H and E stained liver sections were digitally scanned at ×20 magnification. The area of inflammation (µm^2^) per tissue section was determined by a blinded observer using ImageScope. Percentage inflammation was determined by dividing area of inflammation (µm^2^) by area of liver section (µm^2^) × 100.

### Alanine aminotransferase (ALT) liver enzyme analysis

Plasma samples were analyzed at The Mary Lyon Centre, Pathology, Medical Research Council, Harwell, Oxfordshire, UK. A Beckman Coulter AU680 clinical chemistry analyzer (Beckman Coulter, Inc., CA, USA) was used to determine ALT concentrations.

### Statistical analyses

All statistical analyses were performed using GraphPad Prism version 8 software. Statistical tests used for each experiment are described in figure legends.

## Results

### VEGFR2 targeting of therapeutic microbubbles in human colorectal cancer

We first assessed VEGFR2 as a candidate biomarker for tumor blood vessel-specific binding of thMBs in human CRC xenografts. This showed approximately 2.5× more VEGFR2 positive blood vessels in smaller tumors (up to 200 mm^3^) than larger tumors (up to 750 mm^3^) *ex vivo* (**Figure 1B** and**Figure S1A-B**). To relate the immunohistochemical detection of VEGFR2 positive blood vessels with functional VEGFR2 targeting *in vivo*, we used contrast enhanced US with quantitative analysis of VEGFR2 MB binding. Subtracted molecular signals (SMS) equating to the signal from VEGFR2-targeted MBs were higher (but not significantly different) in smaller than larger tumors (11 ± 2.4 versus 6 ± 2.5, respectively, **Figure 1C and Figure S1 C-D**) suggesting that in this model, there was sufficient VEGFR2 across all tumor sizes for it to be actively targeted by thMBs.

To assess the potential for off-target binding, we examined VEGFR2 immunoreactivity in mouse tissues and determined SMS *in vivo*. VEGFR2 was detected in the fenestrated endothelial cells of the liver sinusoids but not in the endothelial cells of the hepatic venules, with weaker staining in kidney cortex and red pulp of the spleen. These areas also demonstrated SMS signals *in vivo* from targeted MB binding (**Figure S1E-F**). Although VEGFR2 is not entirely tumor specific, drug release from thMBs bound to VEGFR2 in non-tumor vessels would potentially be avoided as the US-trigger is localized to the tumor site alone.

thMBs were generated by attaching liposomes to the MB shell (see **Figure S1G** and 43) and VEGFR2 antibody was bound as described in the methods. Under flow conditions *in vitro*, thMBs showed specific binding to VEGFR2 positive cells (**Figure 1D**). Both mouse (SVR) and human (PAE/KDR) VEGFR2 positive endothelial cells bound targeted MBs and thMBs at significantly higher levels (*p* < 0.0001, two-way repeated measure ANOVA) than SW480 CRC cancer cells which showed minimal binding of MBs. Furthermore, there were no significant differences in binding by VEGFR2-targeted MBs alone or thMBs on endothelial cells, confirming that addition of the liposomal payload did not affect targeting efficiency *in vitro*.

We next assessed the ability of thMBs to target a payload *in vivo,* using luciferin as a model liposomal drug for delivery to luciferase-expressing mouse colorectal MC38 xenografts and quantified tumor bioluminescence (BLI) over time 49. Tumor BLI in mice receiving intravenous VEGFR2-targeted luciferin MBs was significantly higher than in those targeted with an isotype-control antibody (**Figure 1E** and**Figure S1G**). Linear regression analysis of the curves showed no difference between the slopes but significant differences in the intercepts (*p* = 0.007) indicating that the rate of release of luciferin from MBs was constant but that there was greater initial delivery of luciferin to the tumor with VEGFR2 targeting and ensuing uptake throughout the tumor due to its highly water soluble nature and the inherent leakiness of the luciferin liposomes. We therefore used VEGFR2-targeting in subsequent experiments to target drug loaded thMBs to the tumor vasculature and investigate US-mediated thMB delivery of cytotoxic drugs* in vivo.*

### Therapeutic microbubbles enhance tumor responses to irinotecan

To determine if thMBs could enhance tumor-specific delivery of a chemotoxic payload *in vivo*, we produced thMBs containing irinotecan (**Figure S2** and**Tables S1A and S1B**). Irinotecan is a member of the camptothecin class of drugs with proven survival benefits as a standard first-line therapy containing fluorouracil-leucovorin-irinotecan (FOLFIRI) with or without bevacizumab in CRC. However, severe adverse effects (diarrhea and neutropenia) often lead to altered dosing schedules or preclude its use 60. We compared tumor drug concentrations, drug metabolism and tumor responses *in vivo* with and without an US-trigger using a bolus of 10^8^ thMBs (intravenous injection volume of the thMBs ranged from 150-200 µL in order to maintain this) consistent with contrast-enhanced US imaging protocols in mice 61, 62. This equated to approximately 2 mg/kg irinotecan per treatment (Tx), 25× lower than the standard therapeutic dose for mice 63.

**Figure 2:**
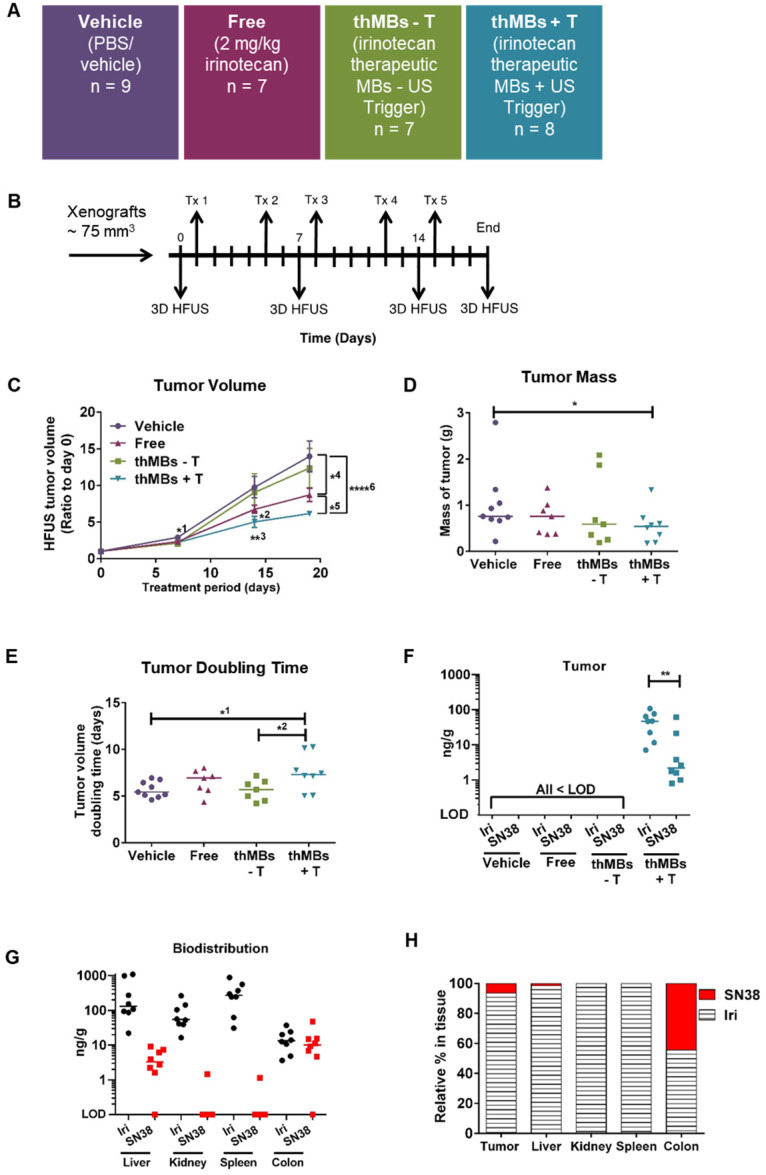
** Therapeutic microbubbles enhance tumor responses to irinotecan**. **(A)** Treatment groups used (*n* = number of mice per group). The abbreviated nomenclature used in subsequent figures is shown. **(B)** Schematic of the experimental protocol is shown with timing of each treatment (Tx) and 3D high frequency ultrasound (HF-US) imaging. The mean (± SEM) starting tumor volumes for each group were 65 (± 7.9) mm^3^ for vehicle, 76 (± 12.5) mm^3^ for free, 72 (± 11.5) mm^3^ for the thMBs -T (no US-trigger) and 85 ± (14.3) mm^3^ for the thMBs + T group which are not statistically significantly different at this time point.** (C)** Effect of thMBs on tumor volume (mean ± SEM) ratio to day 0. Unpaired Mann-Whitney test *^1^ *p* = 0.0152 (vehicle vs thMBs +T), *^2^ *p* = 0.0311 (vehicle vs free), **^3^ *p* = 0.0025 (vehicle vs thMBs +T), *^4^ *p* = 0.0229 (vehicle vs free), *^5^ *p* = 0.0152 (free vs thMBs +T), ****^6^ *p* < 0.0001 (vehicle vs thMBs +T). **(D)** Final tumor mass, unpaired Mann-Whitney test *p* = 0.036 **(E)** Tumor doubling time, unpaired Mann-Whitney test, *^1^ *p* = 0.036, *^2^ *p* = 0.0401. **(F)** Irinotecan and SN38 were only detected by LC-MS/MS analysis in tumors from the thMBs +T group at 72 h post final treatment. LOD indicates the drug was below the limit of detection. **(G)** Irinotecan and SN38 in tissues from the thMB +T group at 72 h post final treatment (median values are indicated). **(H)** Relative amounts of irinotecan and SN38 showing tumor and colon with highest relative levels of conversion of irinotecan to SN38.

The treatment groups and protocols used are shown in **Figure 2A** and** B,** respectively. The customised US-trigger 46 (+T) directed at tumors was a high amplitude, low frequency tone burst with a duration of only 5 s designed to induce MB bursting or cavitation within the tumor 45. This had a Mechanical Index (MI) of 0.21 using the Church approximation 53 and a thermal index in soft tissue of 0.09, both of which are considered safe for all diagnostic US applications. In pilot studies, we showed that this US destruction pulse had no significant effect on the growth of human CRC xenografts (HF-US ratio to day 0 for vehicle was 5.47 ± 1.06 and vehicle +T was 5.76 ± 0.98 (mean, ± SEM; *n* = 4 and 6 per group respectively).

As shown in **Figure 2C,** irinotecan thMBs +T significantly inhibited the tumor growth rate compared with the vehicle control across all imaging time points and with the equivalent dose of free irinotecan on day 18. Tumor mass was also significantly reduced compared to vehicle and tumor doubling time significantly increased (**Figure 2D-E**). The % tumor growth inhibition (%TGI) compared to vehicle (% TGI = 1- (treated final - treated day 0)/ (control final - control day 0) 64) was 38% for free irinotecan and 50% for thMBs + T. In addition, if the free irinotecan is used as the control in the equation, the thMBs +T show a 19% TGI compared to free irinotecan. There was no significant reduction in tumor growth in the thMBs -T group (% TGI -4%) demonstrating that an US-trigger was essential for effective drug release, penetration and/or retention in tumors. Irinotecan-induced biomarker levels changed in all three treatment groups (**Figure S3A-D**) but were significantly enhanced with thMBs +T (tumors showed fewer mitotic cells, increased double strand breaks and apoptotic cells but no change in blood vessel density). Therefore, US-driven thMB delivery apparently increased the potency of irinotecan at these low doses.

To determine if the enhanced tumor responses with thMBs +T were due to altered pharmacokinetics (PK) of drug delivered in this way, we used LC-MS/MS to quantify levels of irinotecan and its active and inactive metabolites (SN38 and SN38 glucuronide [SN38G] respectively) in tumors from all treatment groups, collected 72 h after the final treatment. Irinotecan and SN38 were detected only in tumors from the thMBs +T group (**Figure 2F**) with median (± range) levels of 47 (± 102) ng/g and 2.2 (± 60.4) ng/g tissue, respectively. Both values lie within the effective therapeutic doses *in vitro* (data not shown). No SN38G was detected. Irinotecan and SN38 were below the limit of detection (LOD) in all tumors from the free drug and thMBs -T groups (**Figure 2F**) showing that the US-trigger was required for the enhanced tumor drug accumulation/retention. The extraction efficiencies, stability of compounds and LOD are shown in **Tables S2A-C** and **Figure S4**.

Irinotecan and SN38 were also detected in other tissues of the thMBs +T group (**Figure 2G**). Irinotecan was present in kidney and spleen, with traces of SN38 in a single kidney and single spleen. In the thMBs -T group, irinotecan and SN38 were only detected in colon where 4/7 (57%) had detectable irinotecan and 4/8 mice (50%) had detectable SN38 levels. 1/7 (14%) or 2/7 mice (29%) also had detectable levels of irinotecan or SN38 respectively, in colon in the free drug group (**Figure S5**). This shows that both the free drug and thMBs -T groups had been exposed to irinotecan but that the drug had been metabolised and eliminated and was below the LOD in tumor and liver, indicating an altered PK response with thMBs +T delivery.

We next compared the relative amounts of irinotecan and SN38 in tissues to assess irinotecan metabolism as a surrogate marker of drug release (LC-MS/MS cannot differentiate between liposomally encapsulated or unencapsulated 'free' drugs). Highest relative amounts of SN38 were found in tumor and colon suggesting greater conversion of irinotecan to SN38 in these tissues (**Figure 2H**). In contrast, there were relatively low amounts of SN38 in the liver suggesting limited conversion prior to elimination via hepatobiliary clearance. Persistent liposomal encapsulation of irinotecan and sequestration in cells of the mononuclear phagocytic system (MPS) through which NPs and liposomes are cleared 65-67, may explain this. In rodents, up to 50% of irinotecan is converted to SN38, whereas humans convert less than 8% 68, 69. This suggests that thMB +T targeted delivery of SN38 itself might be more clinically effective.

There was no significant correlation between absolute amounts of irinotecan and SN38 in tumors (Spearman's correlation *r* = 0.48*, p* = 0.3) or livers (Spearman's correlation *r* = 0.59*, p* = 0.13), suggesting non-linear conversion kinetics in both tissues. This may reflect saturable carboxylesterase-converting enzymes or inaccessibility of drug for metabolism due to persistent encapsulation in intact liposomes. By contrast, ratios of irinotecan:SN38 in colon from all groups were close to 1 suggesting a linear conversion rate as indicated by a significant Spearman's correlation (*r* = 0.835*, p* = 0.0007) between individual levels of irinotecan and SN38. This suggests direct conversion of irinotecan to SN38 or deglucuronidation of SN38G to SN38 in the gut microenvironment consistent with bacterial metabolism of irinotecan 70.

### Free irinotecan shows rapid metabolism and excretion *in vivo*

**Figure 3:**
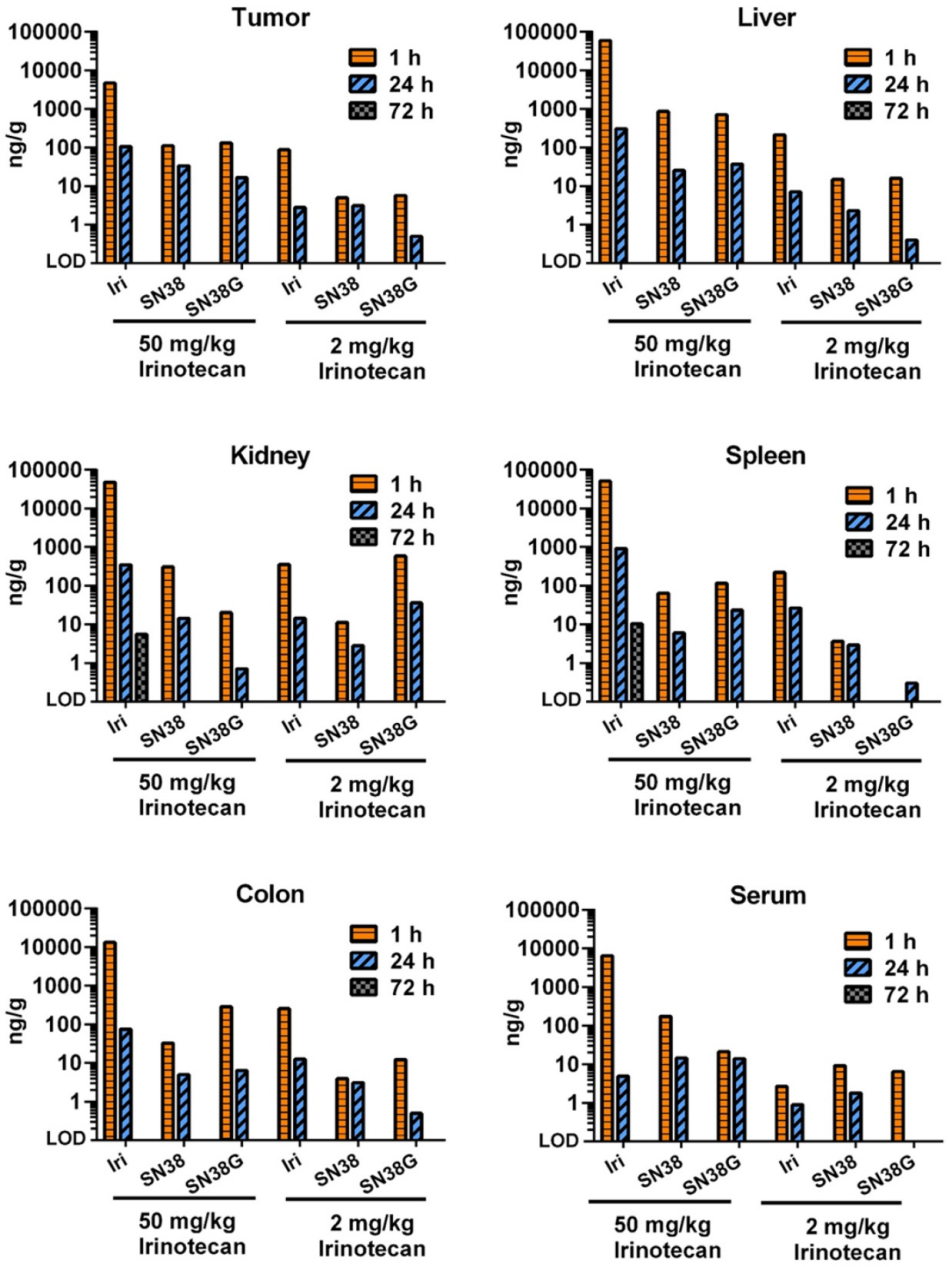
** Free irinotecan shows rapid metabolism and elimination *in vivo***. LC-MS/MS was used to measure the concentration of irinotecan and its metabolites, SN38 and SN38G, in tumors and tissues. High dose (50 mg/kg) and low dose (2 mg/kg) equivalent to that used in thMBs (Figure 2) was delivered intravenously. Free drug was rarely detected after 24 h with only one kidney and one spleen of the three mice in this group showing detectable levels at 72 h using a high-dose of irinotecan. Data from a single injection of irinotecan per time point per mouse are shown except for 72 h where *n* = 3.

We next performed a small study to examine how tumor PK and biodistribution of irinotecan delivered via targeted US-triggered delivery compared with free drug at high dose (50 mg/kg, relevant to routine clinical doses) or at low dose (2 mg/kg, relevant to the dose delivered by thMBs) (**Figure 3**). At 1 and 24 h post-injection of free drug, the levels of irinotecan, SN38 and SN38G were measureable indicating drug delivery and metabolism through to the glucuronidated form was possible in all tissues. The 50 mg/kg dose gave high circulating levels in serum at 1 hour with higher levels in liver, kidney and spleen and the lowest level in tumor. Low dose drug followed a similar pattern with tumor irinotecan levels at 1 hour equivalent to those measured at 72 h post thMB +T administration of the same drug dose. At 72 h post injection of free drug there was no detectable drug in any tissues except for irinotecan in a single kidney and spleen, showing that the altered PK observed with thMBs was not due to the use of a lower dose alone.

### SN38 delivery with US-triggered therapeutic microbubbles significantly inhibits tumor growth

**Figure 4:**
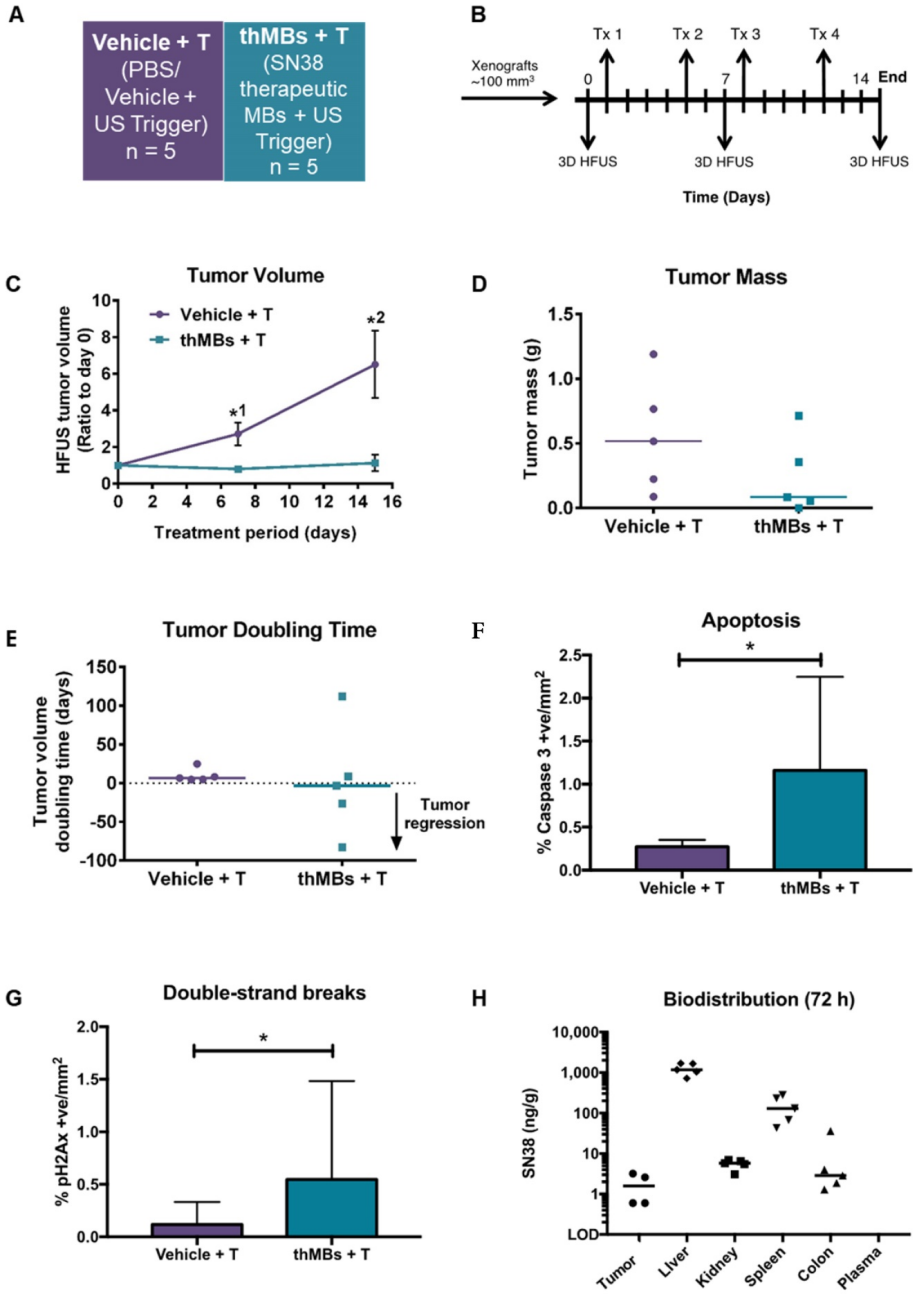
** SN38 delivery with US-triggered therapeutic microbubbles significantly inhibits tumor growth. (A)** Abbreviated nomenclature and treatment groups subsequently denoted in brackets used are shown (*n* = number of mice per group). **(B)** A schematic of the experimental plan is shown with each treatment (Tx, 0.4 mg/kg SN38) and 3D high frequency ultrasound (HF-US) imaging denoted. The mean (± SEM) starting tumor volumes for each group were 100 (± 13.2) mm^3^ for vehicle +T and 106 (± 16.6) mm^3^ for the thMBs +T group which were not statistically significantly different at this time point**. (C)** The mean (± SEM) ratio to day 0 tumor volume measured by 3D HF-US is shown. Unpaired Mann-Whitney test *^1^ *p* = 0.048, *^2^ *p* = 0.0159. **(D - E)** Final day tumor mass was measured and tumor doubling time calculated (negative tumor doubling times indicate tumor regression). The median value for each group is denoted by a line (neither statistically significantly different). (**F - G**) Apoptosis and double-strand breaks**,** the median (+ range) value for each group are denoted. Apoptosis *p* = 0.0159 and double-strand breaks *p* = 0.0317, unpaired Mann-Whitney test. **(H)** Biodistribution of SN38 within all tissues 72 h post final treatment. No SN38G was detected.

To determine if the benefits of thMB delivery in terms of improved PK and tumor responses could be extended to a more cytotoxic molecule, we investigated delivery of SN38 directly as its efficacy at very low doses would be appropriate to the thMB delivery platform. Free SN38 is poorly water soluble with a high partition coefficient (logP 2.65) and cannot be delivered in free drug form without an accompanying high concentration of solvent such as DMSO, thus encapsulation has been the subject of intense research 71-73 and at least one formulation has reached clinical trials 74. SN38 was encapsulated in liposomes and used to formulate SN38 thMBs (**Figure S6** and **Tables S3A-B**). The experimental groups and dosing schedule used for *in vivo* evaluation of SN38 thMBs +T are shown in **Figure 4A** and **B** respectively and were used to deliver 0.4 mg/kg SN38 per Tx.

There was significant inhibition of tumor growth at days 7 and 14 post treatment (**Figure 4C**). Reduced tumor masses and complete regression of one tumor in the thMB +T group were observed (**Figure 4D**). Tumor doubling times were increased or abolished (tumors regressed in volume) in the thMB +T group. Apoptosis and double-strand DNA breaks were also significantly increased as revealed by caspase 3 (**Figure 4F**) and pH2AX staining (**Figure 4G**). The extremely low doses of drug (0.4 mg/kg) delivered by thMBs +T gave 93% TGI. This compared favorably with liposomal SN38 at 8 mg/kg previously shown to give 81-91% tumor growth inhibition in mouse CRC models 75-77.

SN38 was detected in all tumors where sufficient tissue remained at endpoint (4/5) for LC-MS/MS analysis. SN38 was also present at increased levels in other tissues but not in plasma (**Figure 4H**). However, SN38G was not detected suggesting that SN38 remained encapsulated and/or released over time and was below the LOD. This is consistent with irinotecan thMBs +T. ThMB +T delivery increased tumor drug uptake/retention and/or exposure resulting in significant inhibition of tumor growth at a dose 20× less than that given in liposomally encapsulated SN38 studies 75-77.

Irinotecan treatment is often restricted by its dose-limiting side effects. Life-threatening diarrhea, neutropenia and myelosuppression are induced by the active SN38 60. None of the mice from the SN38 thMB +T group (or previous irinotecan thMB groups) exhibited signs of such toxicity (**Figure S7A-E**) suggesting that retention of encapsulated drug in tissues following SN38 thMB +T delivery produced no detectable effects.

### ^89^Zr-labelled SN38 thMBs +T show increased tumor drug accumulation and altered tissue pharmacokinetics compared to VEGFR2-targeted ^89^Zr-labelled SN38 liposomes alone

One strategy to improve liposomal delivery to solid tumors to overcome poor penetration, poor drug potency and inefficient drug release has been to functionalize the surface of the liposome with targeting ligands including peptides, antibodies or aptamers and several actively targeted formulations are in clinical trials 78.

**Figure 5:**
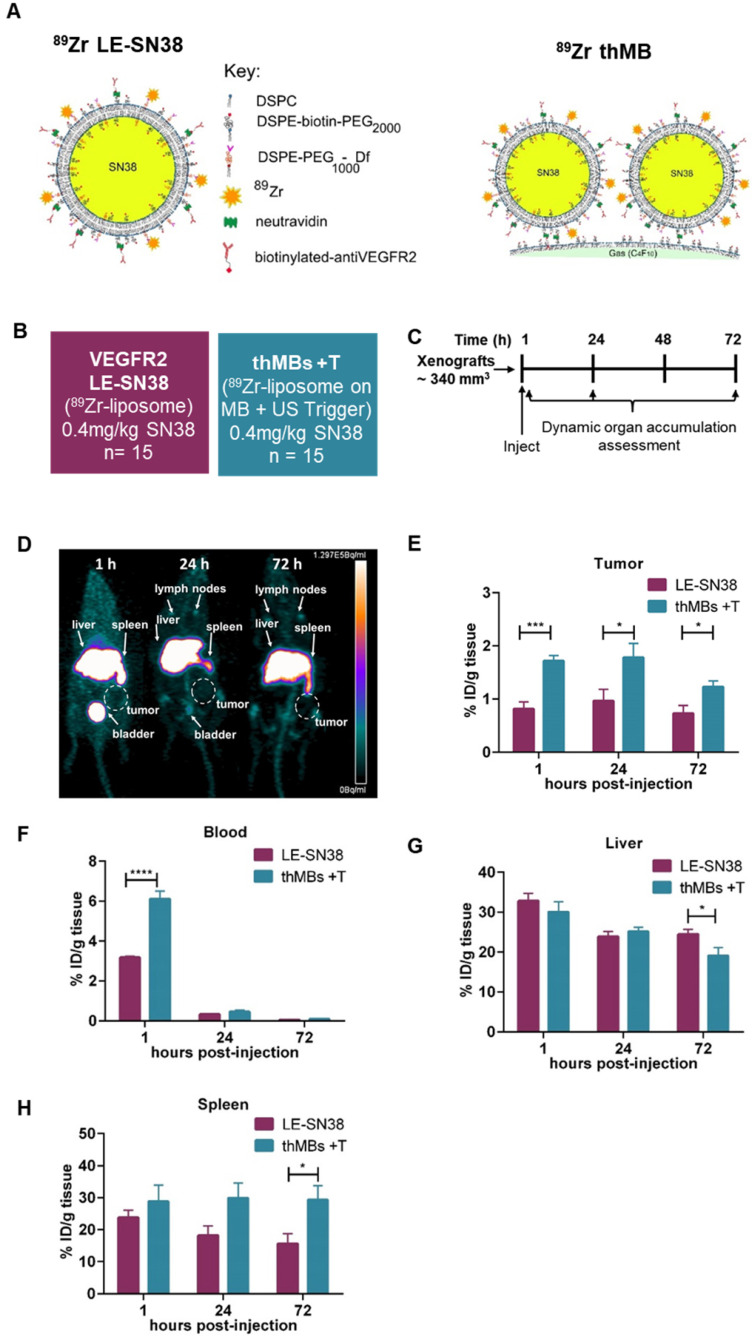
** Improved tumor accumulation of ^89^Zr-labelled SN38 thMBs +T compared with VEGFR2-targeted ^89^Zr-labelled SN38 liposomes alone. (A)** Schematic depicting the strategy used for radiolabeling of SN38 liposomes and SN38 thMBs. DSPE was radiolabeled with ^89^Zr and incorporated into the lipid shell of liposomes encapsulating SN38 and used to generate therapeutic MBs. **(B)** Schematic of the experimental groups used to compare VEGFR2-targeted liposomally encapsulated SN38 (LE-SN38) with SN38 thMB +T (*n* = number of mice per group). **(C)** Schematic of the experimental protocol used (*n* = 5 animals per time point). The mean (± S.D.) starting tumor volume measured by mechanical calipers was 340 mm^3^ ± 134 mm^3^. **(D)** PET image from the thMB +T group at each imaging time point. Signal in the liver, spleen and bladder are arrowed, tumor is circled. **(E)** The percentage injected dose per gram of tissue (%ID/g tissue) in tumor **(F)** blood **(G)** liver and **(H)** spleen were calculated with mean and SEM shown. Unpaired T-test tumor, ***p = 0.0006 at 1 h, *p = 0.0432 at 24 h and *p = 0.026 at 72 h. Blood ****p < 0.0001 at 1 h, liver *p = 0.0494 at 72 h, spleen *p = 0.0326 at 72 h.

To directly compare targeted delivery by thMBs with a clinically relevant nanoformulation, we carried out ^89^Zr-radiolabelled distribution studies with longitudinal PET imaging in mice. Mice bearing human CRC xenografts were used to examine biodistribution and quantitate uptake of VEGFR2-targeted ^89^Zr-SN38 liposomes alone compared with ^89^Zr-SN38 thMBs +T (depicted in **Figure 5A**). Radiolabelling of pre-formed liposomes incorporating a deferoxamine chelator was necessary as SN38 itself was not amenable to direct labelling with ^89^Zr due to its relatively small size. Treatment groups and protocols used are shown in **Figure 5B** and **C** respectively.

PET images at 1, 24 and 72 h post intravenous injection with ^89^Zr-SN38 thMBs +T (**Figure 5D**) showed a similar distribution pattern to VEGFR2-targeted ^89^Zr-SN38 liposomes alone, with the majority of the signal detected in liver. Signal was also evident in spleen and lymph nodes suggesting clearance predominantly through the MPS system, as previously described for liposomes 65-67 and VEGFR2-targeted MBs 79.

*Ex vivo* radiocounting was used to quantitate the percentage injected dose in different tissues (%ID/g tissue) over time. This showed significantly increased (approximately twice as much) ^89^Zr in tumors at all time points in the ^89^Zr-SN38 thMBs +T group compared with VEGFR2-targeted ^89^Zr-SN38 liposomes alone (**Figure 5E**). There was also significantly increased signal from thMBs in blood at one hour post injection compared to free liposomes which declined to similar levels by 24 h (**Figure 5F**) suggesting enhanced retention and circulation of thMBs in the vascular space compared with targeted liposomes alone. By 72 h post-injection, there was a significant difference in %ID/g tissue in liver and spleen, with relatively less in liver and more in spleen in the ^89^Zr-SN38 thMBs +T group. There was also a difference in the ratio of %ID/g in liver and spleen compared to tumor (**Figure S8B-C**) with a significant 3-fold decrease in liver to tumor ratio %ID at all time points.

## Discussion

We show here how thMBs can enhance the efficacy of cytotoxic drugs *in vivo* by increasing the concentration of encapsulated drug in the circulation, increasing tumor drug accumulation/retention and limiting bioavailability and toxicity in normal tissues. Sensitive LC-MS/MS quantification of drugs and their metabolites showed that thMBs limited exposure in normal tissues by persistent encapsulation of the drug, consistent with uptake by the MPS in tissues not exposed to US. The extended therapeutic range appears to be due to multiple factors that contribute to altered drug distribution, metabolism and route of elimination, thereby achieving significant therapeutic responses with very low doses of cytotoxic drugs. The different payloads used in this study demonstrate the flexibility and ease of use of a microfluidic production platform for generating thMBs in a simple 'one-pot' approach, with potential for scale-up and a route to clinical grade production for human trials.

These data show that thMB efficacy *in vivo* can be attributed to the combination of several different factors. VEGFR2 provided a tumor vasculature target for the accumulation, binding and/or anchoring of thMBs within the tumor microenvironment. Like others 31, 80, we found this interaction to be specific compared to control antibodies or non-VEGFR2 expressing cells and correlated with expression of VEGFR2 in tumors. Using VEGFR2 targeting to tumor endothelium, thMBs potentially increase the number of MBs and liposomes tethered in close proximity to the vessel walls. It is this proximity which presumably aids intra- and/or intercellular drug delivery as VEGFR2-targeted MB delivery of luciferin showed higher initial uptake of luciferin in tumors in the absence of an US-trigger.

The US-trigger was shown to be essential for the selective effects of VEGFR2 targeted thMB delivery of stably encapsulated irinotecan as without the trigger, thMBs failed to extend intra-tumoral drug delivery or demonstrate enhanced tumor responses. The effects of the 5 s US-trigger were long-lasting and widespread as shown by the extended drug retention in tumors and also in tissues at 72 h. Zhang *et al*. 25 also showed that an US-trigger enhanced tumor responses more than endothelial targeting (via RGD) in breast cancer xenografts, using liposome-loaded MBs, although they were additive in effect. Their study used a longer US pulse and a much higher dose of paclitaxel than used here to generate a similar therapeutic effect 25.

US-triggered thMB drug targeting to tumors can therefore potentially be achieved on two levels. Firstly, molecular interaction occurs with a biological receptor on the tumor endothelium (VEGFR2). Secondly, physical targeting or site-specific release is promoted locally by the external delivery of an US-trigger, directed at the bound and flowing (unbound) thMBs within the tumor microenvironment. Together, these increase tumor cell uptake and/or retention of drug as seen with irinotecan thMBs. In model systems (27), targeting of MBs to a surface and exposure to US showed drug release and enhanced transport consistent with the *in vivo* effects observed here.

In addition to VEGFR2 targeting and a localized US-trigger, the MB structure itself was shown to play an important role in enhancing drug delivery since PET radiotracing of thMBs showed a two-fold increased concentration in blood compared with targeted liposomes alone at 1 hour post US-trigger. Our extensive analysis of tumor as well as tissue pharmacokinetics, provides insight as to how the thMB structure and the US trigger impacts on drug distribution, retention and elimination and enhances the effects of very low dose thMB delivery compared with systemic delivery of free irinotecan or VEGFR2 targeted SN38 liposomes.

The mechanism of US-mediated drug delivery is being actively studied *in vitro* using a variety of techniques including very high-frame rate microscopy. The Brandaris 128 ultra-high-speed-camera combined with confocal microscopy has been used to study MB oscillations and MB destruction under different US parameters as well as free drug delivery to cells 81. Roovers *et al*. sectioned spheroids incubated with drug-loaded MBs and exposed to US and found that lipids from the liposomes were 'sonoprinted' onto the surface of the spheroids following which drug release into deeper regions was measured 82. Recently published work from our group showed a delay in drug uptake in spheroids using drug-loaded MBs compared to free drug co-delivered with microbubbles 83 suggesting that the attached liposomes were intact following US-mediated MB destruction and prior to cellular uptake. However, further high-speed two-color camera interrogations will be required to elucidate the mechanism in real time.

Several groups have demonstrated the importance of the liposomal attachment to MBs 23, 84, 85 and direct linkage of drug loaded liposomes has been shown to improve drug delivery compared to co-delivery with free drug 23, 86. US destruction of untargeted DOX-loaded MBs has previously been shown to enhance localized drug delivery, although the mechanisms were not determined 87. The local energetic stimuli arising from US destruction of multiple attached and flowing thMBs in the tumor vasculature may involve physical forces (radiation, acoustic) jetting or microstreaming 88. Theoretical and single MB experiments *in vitro* have suggested that oscillation of MBs may shed the liposomal payload. However, our study uses a short MB destruction pulse which is highly unlikely to cause MB oscillation alone. The thMB construct itself and the fragmentation products arising from US-triggered destruction may well underlie the altered PK properties of encapsulated drugs delivered in this way. It has been shown that in the absence of an ultrasound trigger, thMBs delivering a fluorophore could be imaged in tumors from 4 h post-injection, whereas with a trigger this accumulation occurred from the earliest imaging time point (0.5 h), irrespective of whether molecular targeting (iRGD) was present 89.

The efficacy of thMB delivery depends on MB design (e.g. size, shell properties, stability) as well as the drug itself. We initially chose irinotecan as despite its clinical application, the incidence of severe diarrhea and neutropenia can limit its use. Furthermore, the unpredictable toxicity (despite genotyping for high risk glucuronidation deactivating enzyme polymorphic variants e.g. UGT1A1 *28/*28) is an increasing cause for concern 60. Although it has been successfully encapsulated and liposomal formulations have shown reduced toxicity 90, localized thMB delivery enables much lower systemic doses to be used and reduces the potential for dose-limiting toxicities even further. We also encapsulated SN38 for direct thMB delivery as it is up to 1000× more toxic than irinotecan and demonstrates the applicability of this platform for administration of difficult to deliver drugs. In addition, the conversion to active forms of irinotecan/SN38 (lactone) are favored in the more acidic tumor microenvironment and this may also contribute to the reduced toxicity seen in healthy tissues at physiological pH (where it is very rapidly converted to the carboxylated form 70) as well as the enhanced TGIs seen with such low doses.

In traditional systemic dosing, the effectiveness of chemotherapy is determined by the tumor drug concentration and high circulating concentrations are required to obtain a therapeutic response. Liposomal formulations have the benefit of prolonged blood circulation time resulting in longer half-life, larger AUC, and slower clearance, but suffer from unspecified timing and location of drug uptake. Preclinical activity of liposomal Irinotecan has been shown to be governed by tumor deposition and intratumor prodrug conversion. A mechanistic PK model showed that concentration and time of exposure were major determinants of irinotecan efficacy in liposomal formulations and tumor permeability and carboxylesterase function limited liposomal irinotecan delivery to tumors 64. As shown here, thMBs provide a route to achieving efficacy and overcoming these potential barriers to drug delivery in the context of irinotecan and SN38. With thMB delivery, drug concentrations delivered in a single bolus were 2 mg/kg for irinotecan and 0.4 mg/kg for SN38, far lower than those usually used systemically for irinotecan 63, 69 or for liposomal irinotecan (3.75 mg/kg) 64 and up to 8 mg/kg with liposomal SN38 75-77. Although we did observe some heterogeneity of response in the treatment groups, further much larger studies would be required to investigate this. However, statistically these results showed that the thMB response does not require high circulating levels of drug in plasma to achieve therapeutic effects in tumors.

ThMB delivery may prove particularly effective in some clinical settings. For example, the increasingly elderly population or those living with cancer would benefit from the low drug exposures that allow repeated delivery. In addition, there is utility for treating recurrent abdominal disease as well as metastatic CRC and hepatocellular carcinoma since the drug remains encapsulated in normal liver. Precisely focused US exposure would be necessary to achieve this.

Molecular imaging of VEGFR2 expression in patients has been proposed for antiangiogenic therapies 91 and may facilitate stratification of patients for thMB treatment. This could be achieved with contrast-enhanced ultrasound or multimodal imaging. With nearly 60 clinical trials involving MBs currently listed as open on the NIH Clinical Trials database, including 3 for treatment of gastrointestinal cancers, there is ongoing interest in the field of MBs. To facilitate translation of thMBs to the clinic, further optimization of the approach is warranted for example, by investigating alternative US parameters such as a higher MI to target central tumor areas 89 or fractionated and longer US pulses than were used in this study or a focused US beam to limit any potential for off-target US triggering. Freeze-drying of our drug loaded thMBs has also been achieved (data not shown) which will provide a facile way to clinical usage as well as aiding production at scale. The use of alternative targeting strategies such as affimers 92 or lipopeptides 93 should also be explored to reduce potential costs of treatment. Further evaluation of different liposome payloads for thMBs, thMB manufacture and theranostic clinical US systems will also aid more rapid translation of this technology for clinical use.

## Abbreviations

ADMET: absorption, distribution, metabolism, elimination and toxicity
US: ultrasound
BLI: bioluminescent imaging
CRC: colorectal cancer
DF: deferoxamine mesylate
DPPC: 1,2-dipalmitoyl-sn-glycero-3-phosphocholine
DSPC: 1,2-distearoyl-sn-glycero-3-phosphocholine
DSPE-B-PEG_2000_: 1,2-distearoyl-sn-glycero-3-phosphoethanolamine-N-[biotinyl (polyethylene-glycol)-2000]
EPR: enhanced permeability and retention effect
FCS: fetal calf serum
FOLFIRI: fluorouracil-leucovorin-irinotecan
HF-US: high-frequency ultrasound
HPLC: high-performance liquid chromatography
IHC: immunohistochemistry
Iri: irinotecan
LC-MS/MS: liquid chromatography tandem mass spectrometry
LOD: limit of detection
MI: mechanical index
MBs: microbubbles
MPS: mononuclear phagocytic system
NPs: nanoparticles
%ID/g: percentage injected dose per gram of tissue
PK: pharmacokinetics
PBS: phosphate buffered saline
pH2AX: phosphorylated histone H2AX
PET: positron emission tomography
SEM: standard error of the mean
SN38G: SN38 glucuronide
SMS: subtracted molecular signal
Texas Red® DHPE: Texas Red® 1,2-dihexadecanoyl-sn-glycero-3-phosphoethanolamine, triethylammonium salt
TGI: tumor growth inhibition
thMBs: therapeutic microbubbles
+/-T: with or without an ultrasound trigger
Tx: treatment
US: ultrasound
VEGFR2: vascular endothelial growth factor receptor 2
Zr: zirconium
