# Supplementary material for: Ultrasound-triggered therapeutic microbubbles enhance the efficacy of cytotoxic drugs by increasing circulation and tumor drug accumulation and limiting bioavailability and toxicity in normal tissues
Source: Theranostics. 2020 Sep 1;10(24):10973–92. doi: 10.7150/thno.49670 (PMC7532679; doi:10.7150/thno.49670)
Supplement: Supplementary file 1 — Supplementary figures and tables. [file thnov10p10973s1.pdf]

SUPPLEMENTARY MATERIAL:

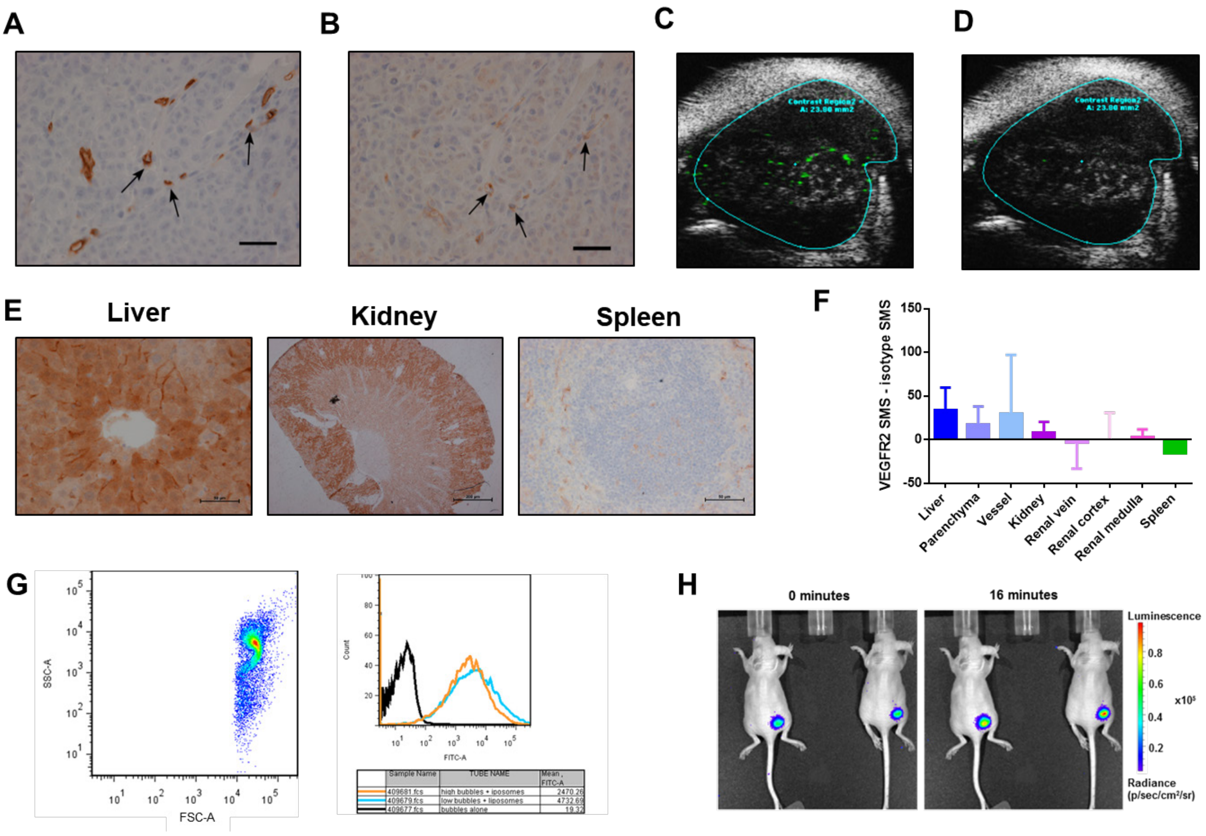

Figure S1 VEGFR2 targeting of therapeutic microbubbles in human colorectal cancer. HCT116 CRC xenografts were immunostained for **(A)** CD31 and **(B)** VEGFR2. Arrows denote examples of positively stained endothelial cells. The scale bar denotes 50 μm. See **Figure 1B** for VEGFR2/CD31 double positive blood vessels in relation to tumor size. Contrast enhanced ultrasound of the tumor (outlined in cyan) showing VEGFR2-targeted MBs pseudocolored in green before **(C)** and after **(D)** a destruction pulse is applied. **(E)** VEGFR2 positivity in sinusoids in liver (scale bar = 50 μm), in the cortex and to a lesser extent the medulla in the kidney (scale bar = 200 μm) and in the spleen (scale bar = 50 μm). **(F)** SMS signal from VEGFR2 or isotype-targeted Micromarker in these organs quantified in different regions with the same size ROI, mean +/- S.D. shown of *n* = 3 mice except for spleen where *n* = 1. **(G)** Flow cytometry of MBs (left) which was used for gating in the histograms (right). Liposomes were labelled with a green fluorescent lipid and showed a 2-log shift in fluorescence of the MB population when bound (two different concentrations of

MB were flowed through the machine, high = 10^7/mL and low = 10^6/mL. **(H)** An example of bioluminescent imaging of mice injected with either isotype-targeted luciferin MBs (left) or VEGFR2-targeted luciferin MBs (right). See **Figure 1E** for graph average radiance over time.

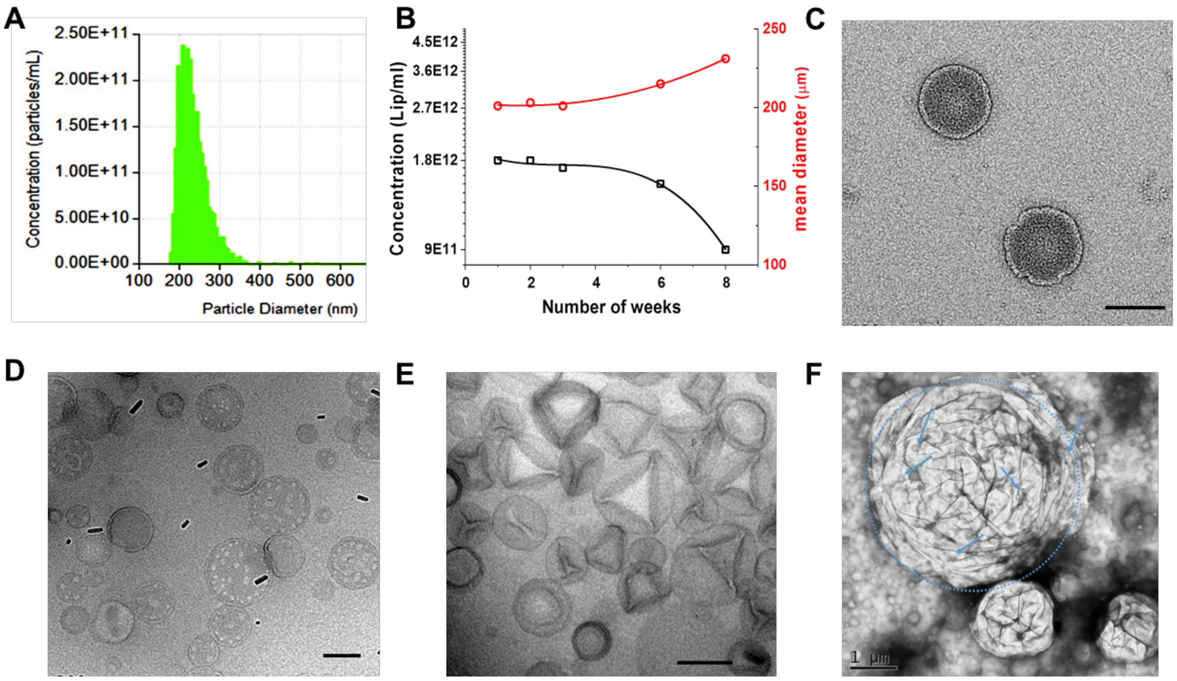

Figure S2. Characterization of irinotecan liposomes and thMBs. **(A)** Q-nano histogram for liposome concentration and size distribution. **(B)** Liposome stability at 4 °C as a function of size and concentration versus time in weeks. **(C - E)** Transmission electron microscopy (TEM) images for liposome production at each stage; empty liposomes **(C)**, liposomes containing ionophore **(D)** and liposomes with encapsulated irinotecan **(E)**. Scale bar denotes 200 nm **(C - E)**. **(F)** TEM image of a thMB, the largest MB is outlined in blue and some of the attached liposomes are arrowed. Scale bar denotes 1 μm.

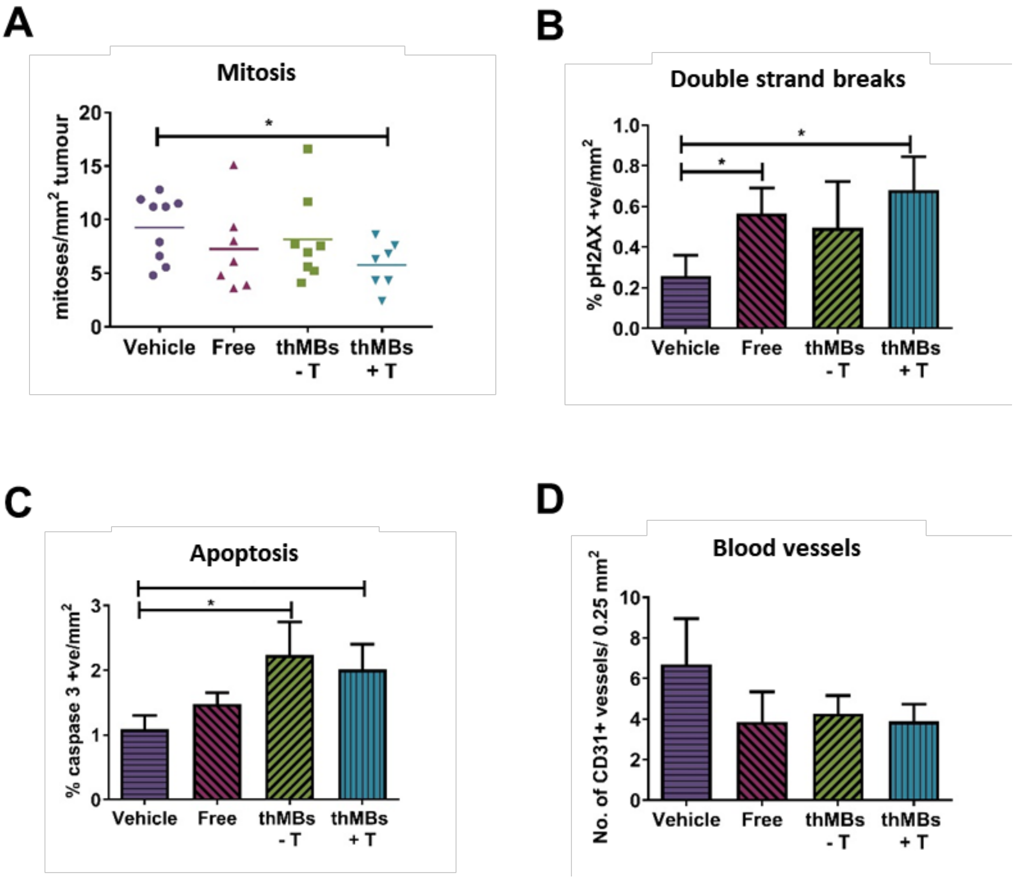

Figure S3. Pharmacodynamic responses of tumors to irinotecan delivered by thMBs. **(A)** Numbers of mitoses per mm^2 of tumor tissue. The median value for each group is denoted in **(A - D)** (**B**) Double-strand breaks **(C)** apoptosis **(D)** number of vessels per 0.25 mm^2 of tumor (mean (± SEM; *p < 0.05, Mann-Whitney, U-test, two-tailed).

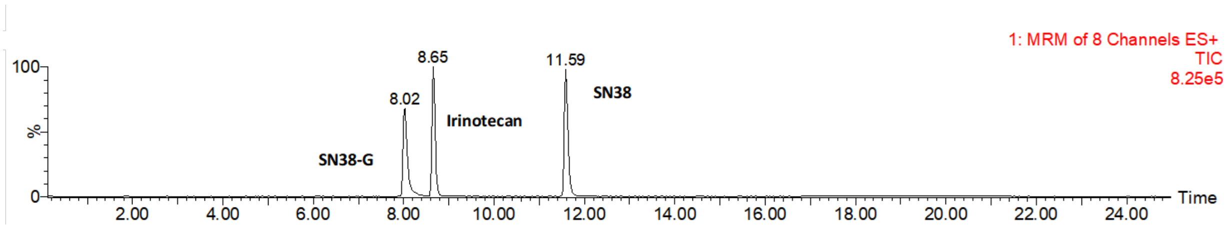

Figure S4. Chromatograms of Irinotecan, SN38 and SN38G with Detection by LC-MS/MS and Limits of Detection. Example chromatogram of Irinotecan, SN38 and SN38G (1 μg/mL standard, 10 μL injection). Retention time for SN38G is approximately 8 min, for Irinotecan 8.7 min and for SN38 11.5 min. To determine the lower limits of detection, decreasing amounts of a 10 ng/mL solution of irinotecan or SN38 in methanol were examined by LC-MS/MS. 10 pg/L could be detected above background.

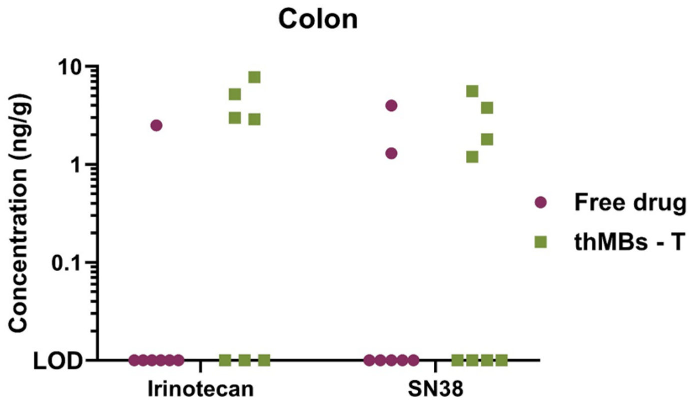

Figure S5. LC-MS/MS detection in colon. The presence of irinotecan and SN38 was quantitated in all treatment groups (see **Figure 2G** for thMBs +T). Irinotecan was detected in 1/7 of the mouse colons in the free drug group and 4/8 of the mouse colons in the thMBs – T group. SN38 was detected in 2/7 of the mouse colons in the free drug group and 4/8 of the mouse colons in the thMBs –T group.

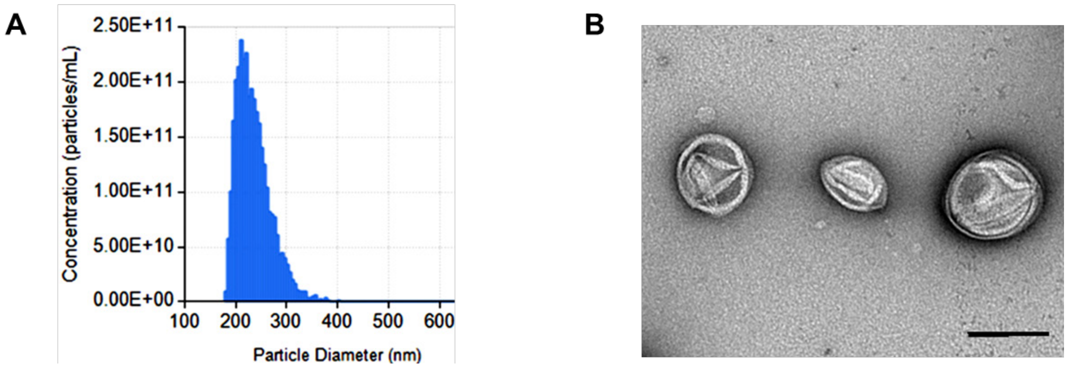

Figure S6. Characterisation of SN38 liposomes. (A) Q-nano histogram for liposome concentration and size distribution. **(B)** Transmission electron microscopy (TEM) image of liposomes with encapsulated SN38. Scale bar denotes 200 nm.

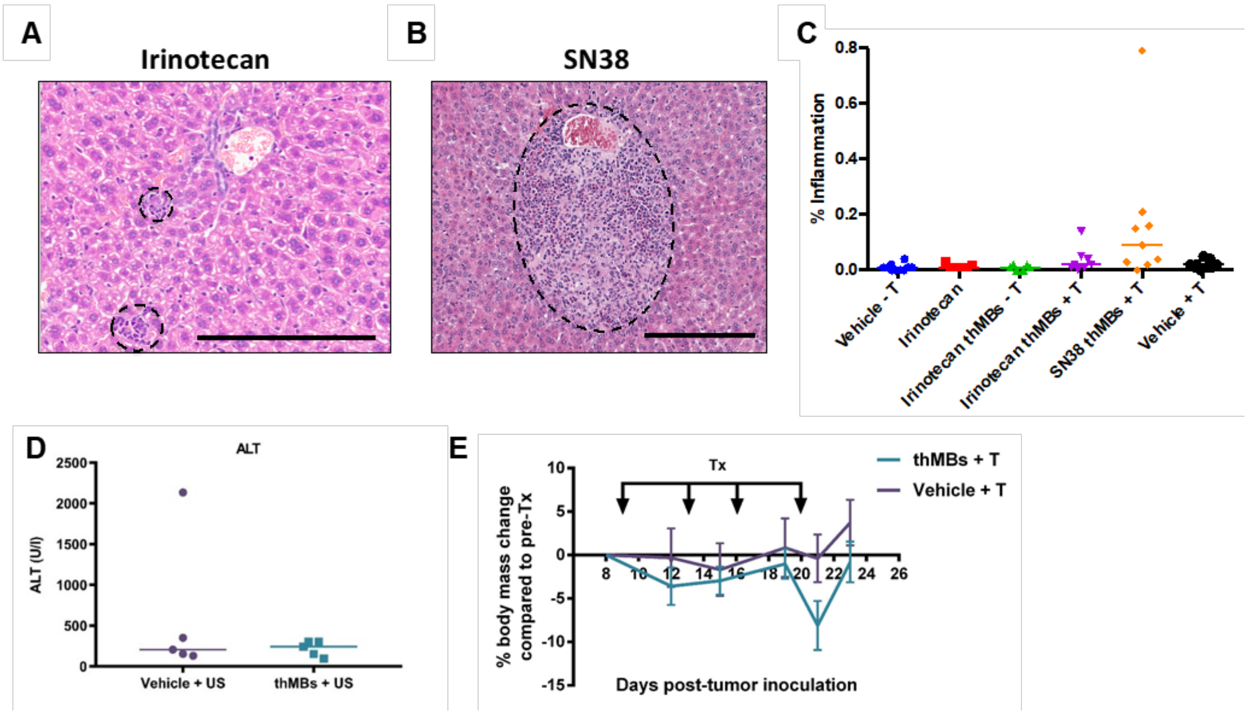

Figure S7. Tolerability of thMBs treatment. Hematoxylin and eosin-stained sections of liver treated with Irinotecan **(A)** and SN38 **(B)** thMBs +T respectively. The dotted line surrounds foci of mild perivascular inflammation adjacent to a blood vessel, scale bar denotes 200 µm. **(C)** Percentage perivascular inflammation in the liver of all treatment groups. The median value is shown. **(D)** Plasma alanine amino transferase (ALT) levels

were measured and no difference was observed. The percentage change in body mass from pre-treatment levels was plotted, treatment days (Tx) are indicated. No statistically significant difference was observed between treatment groups **(E)**.

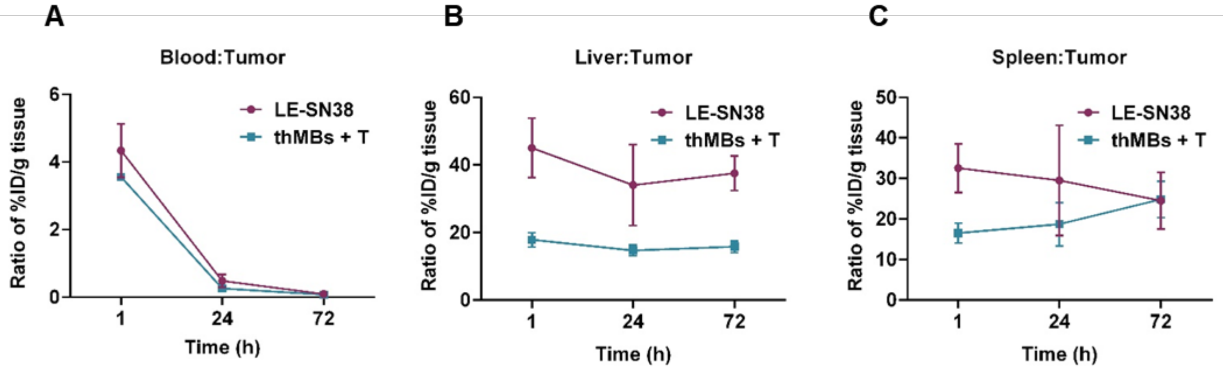

Figure S8. Reducing delivery to non-target organs. The ratios of the %ID/g of tissue were plotted from the ^{89}Zr-labeling experiment (see **Figure 4**). **(A)** The ratio blood:tumor between the LE-SN38 and the thMB +T group. **(B)** The ratio between liver:tumor between the LE-SN38 and the thMB +T group. **(C)** The ratio between spleen:tumor between the LE-SN38 and the thMB +T group. Mean ± SEM are shown.

A

Characteristics irinotecan liposome preparations			
Liposome prep	Concentration (x 10^{12} liposomes/ml)	Mean diameter (nm)	Irinotecan concentration (mg/ml)
1	1.8	201	1.86
2	2.6	196	3.23
3	1.5	221	2.02
4	0.5	245	1.09
Mean	1.6	215	2.05
Std Dev	0.9	22	0.89

B

Characteristics thMB preparations			
Microbubble prep	Concentration (x 10^{8} MBs/ml)	Mean diameter (μm)	Irinotecan concentration (mg/ml)
1	7.0	1.4	N.D.
2	6.9	1.4	N.D.
3	7.6	1.5	N.D.
4	6.0	1.7	0.33
5	5.4	1.7	0.47
6	3.6	2.4	0.18
7	2.5	2.5	0.23
8	7.1	1.8	0.23
9	4.7	1.9	0.23
10	8.3	1.8	0.18
11	9.2	1.6	0.21
Mean	6.2	1.8	0.26
Std Dev	2.0	0.4	0.10

Table S1. The characteristics of **(A)** the irinotecan liposome preparations and **(B)** thMBs used in the study. Four preparations were required to provide enough liposomes for all the treatment groups. These were sized and counted using a Q-Nano and the irinotecan content measured by HPLC. Eleven preparations of thMBs were required to inject all the groups at each time point. The concentrations of MBs and their mean diameter were measured by light microscopy. The irinotecan content was measured by HPLC. The first three preparations used for injection did not have enough remaining for HPLC analysis, therefore the concentration of irinotecan was not determined (N.D.).

A

Compound specific mass spectrometer MRM settings

Compound	Precursor/product ion (m/z)	Dwell (sec)	Cone voltage	Collision energy (volts)
Tolbutamide	271.2 > 155.0	0.20	20	15
Tolbutamide	271.2 > 172.0	0.20	20	15
SN38	393.2 > 264.2	0.15	35	30
SN38	393.2 > 293.0	0.15	35	30
SN38	393.2 > 349.1	0.15	35	30
SN38-G	569.8 > 393.8	0.20	35	30
SN38-G	569.8 > 349.9	0.20	35	30
Irinotecan	587.3 > 124.0	0.15	25	45
Irinotecan	587.3 > 167.0	0.15	25	45
Irinotecan-d10	597.3 > 133.1	0.15	25	45
Irinotecan-d10	597.3 > 177.1	0.15	25	45

B

Extraction efficiency of irinotecan, SN38 or SN38-G

	Irinotecan (PA)	SN38 (PA)	SN38-G (PA)
Tumour	43932	57047	50154
MeOH	50603	53389	46403
Extraction (%)	86.8	106.9	108.1
Liver	53343	55450	49667
MeOH	47623	48223	41672
Extraction (%)	112.0	115.0	119.2
Kidney	54896	68279	61335
MeOH	56386	59948	51212
Extraction (%)	97.4	113.9	119.8
Colon	47216	55290	52865
MeOH	45143	45804	39355
Extraction (%)	104.6	120.7	134.3
Serum	53519	60195	55351
MeOH	51961	52740	44508
Extraction (%)	103.0	114.1	124.4

C

Stability of compounds at 8°C over 18 hours

Time (h)	Irinotecan (PA)	SN38 (PA)	SN38-G (PA)
0	44709	50829	42720
2	46807	56498	50972
4	38685	43927	37376
6	40024	51081	49105
8	33831	38921	31589
10	43948	49133	42842
12	45926	51760	43129
14	49351	61483	58751
16	49178	54388	45870
18	37409	52597	46142
Average	42987	51062	44850
Std Dev	5251	6273	7415
% CV	12.2	12.3	16.5

Table S2 LC-MS/MS Parameters. **(A)** The instrument settings used to detect the analytes during each run are shown. **(B)** Extraction efficiency of irinotecan, SN38 and SN38G in tissues, compared to 100% methanol, by LC-MS/MS. Tissue homogenates were spiked with 1 µg/mL of irinotecan, SN38 or SN38G, and compared to 100% methanol spiked in the same way. Peak area (PA) is given and shows good extraction efficiency in tissues compared to the same dose dissolved directly in methanol. N.B. >100% extraction is due to the reduced volume after the protein has been pelleted from the homogenate and shows none was bound to this protein fraction. **(C)** Stability of irinotecan, SN38 and SN38G at 8 ^{o}C for 18 h by LC-MS/MS. 1 µg/mL of irinotecan, SN38 or SN38G were analyzed every 2 h by LC-MS/MS to look at their stability within the sampling chamber prior to injection. PA values for each are shown.

A Characteristics SN38 liposome preparations

Liposome prep	Concentration (x 10^{12} liposomes/ml)	Mean diameter (nm)	SN38 concentration (mg/ml)
1	4	242	179.3
2	4	242	255.5
3	4	242	299.5
4	4	242	209.5
5	4	242	224.2
Mean	4	242	233.6
Std Dev	0	0	41.1

B Characteristics SN38 thMB preparations

Microbubble prep	Concentration (x 10^{8} MBs/ml)	Mean diameter (μm)	SN38 concentration (mg/ml)
1	7.8	1.6	11.5
2	6.5	2.2	11.7
3	4.4	1.9	13.6
4	5.9	1.8	10.6
5	3.7	1.7	10.8
Mean	5.6	1.8	11.6
Std Dev	1.7	0.2	1.2

Table S3. The characteristics of **(A)** the SN38 liposome preparation (a single large batch was made and freeze-dried in aliquots) and **(B)** thMBs used in the study. For each of the five treatments, one aliquot of liposomes was rehydrated and used to generate thMBs. Liposomes were sized and counted using a Q-Nano on a single separate aliquot but the SN38 content in both the liposomes and thMBs was measured by HPLC from a small sample retained for this purpose at each Tx. The concentrations of MBs and their mean diameter were measured by light microscopy.
